# Highly Chiral MoS_2_ Flakes With Redox‐Active Centers for Spin Selective Water Oxidation

**DOI:** 10.1002/smsc.70356

**Published:** 2026-08-17

**Authors:** Anastasiia Tulupova, Denis Zabelin, Ivan Kirishiantsev, Ruslan Ramazanov, Rashid Valiev, Tomas Hrbek, Lucia Bajtosova, Miroslav Cieslar, Alena Michalcova, Vladislav Buravets, Vasilii Burtsev, Marie Urbanova, Vaclav Svorcik, Oleksiy Lyutakov

**Affiliations:** ^1^ Department of Solid State Engineering University of Chemistry and Technology Prague Czech Republic; ^2^ Department of Chemistry University of Helsinki Helsinki Finland; ^3^ Department of Surface and Plasma Science Faculty of Mathematics and Physics Charles University Prague Czech Republic; ^4^ Faculty of Mathematics and Physics Charles University Prague Czech Republic; ^5^ Department of Metals and Corrosion Engineering University of Chemistry and Technology Prague Czech Republic; ^6^ Department of Physics and Measurements University of Chemistry and Technology Prague Czech Republic

**Keywords:** chiral nanomaterials, chiral‐induced spin selectivity, MoS_2_, Ni doping, water oxidation

## Abstract

We propose a hydrothermal synthesis route for highly chiral two‐dimensional flakes of MoS_2_ doped with single nickel atoms (Ni:MoS_2_) for spin‐controlled water oxidation. Organic enantiomers were added to the reaction mixture as chiral encoders. The resulting MoS_2_ material obtained in the 1T phase exhibited wide absorption bands throughout the visible spectrum. Circular dichroism measurements indicated a pronounced chiroptical response at both UV and visible wavelengths. After delamination, the chirality was retained, and the Ni:MoS_2_ flakes were subsequently tested for water oxidation under the assumption that the flakes could support chiral‐induced spin alignment of the transiting electrons. The chiral‐induced spin selectivity (CISS) effect facilitates water oxidation kinetics and tunes the reaction selectivity toward oxygen production. The results of the electrochemical experiments, subsequently confirmed by density functional theory calculations, showed a significant decrease in the water‐oxidation offset and onset potentials for the optimal combination of doping and flake chirality. In turn, the CISS effect manifests itself as an apparent suppression of hydrogen peroxide production and enhancement of oxygen production. Our work provides clear evidence of how chiral transition metal dichalcogenides can be used to tune the reaction efficiency and selectivity in energy‐related applications.

## Graphical abstract

The chiral‐induced spin selectivity effect was realized exploited in Ni‐doped, intrinsically chiral 2D MoS_2_ flakes to suppress H_2_O_2_ production and enhance water oxidation kinetics during water splitting.

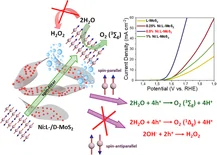

## 1 Introduction

Control over the kinetics and pathways of chemical reactions is commonly achieved by varying several parameters, such as catalyst addition, temperature, and solvent, as well as illumination in the case of photochemistry or the application of a bias voltage in electrochemistry [1, 2, 3, 4, 5, 6]. Recently, an alternative method for influencing reaction selectivity and efficiency was introduced based on the spin selectivity of the transferred electron current [7, 8]. In this case, the alignment of electron spins can support or suppress the electronic configuration of the reaction products or intermediates and thereby alter the reaction activation barrier and final product ratio [9]. In pioneering studies, the concept of spin‐determined reaction kinetics was realized using circularly polarized light or an external magnetic field [10, 11, 12]. More recent studies have shown that spin selectivity can be achieved with chiral nanomaterials using spin filtering or spin polarization during electron transport through these materials (the so‐called chiral‐induced spin selectivity (CISS) effect) [8, 13, 14].

The CISS effect was initially demonstrated using a layer of chiral organic molecules deposited on a suitable conductive and/or redox‐active surface [15, 16, 17]. The recent development of chiral materials exhibiting the CISS has shifted toward the creation of organic–inorganic hybrid systems, which possess a higher degree of spin polarization [18]. Among these materials, conjugated polymers, hybrid perovskites, metal–organic frameworks, and transition metal dichalcogenides (TMDC) have been reported [19, 20, 21, 22, 23, 24, 25, 26]. In the last case, chiral TMDC were prepared using different techniques, including intercalation of the chirality encoder (organic enantiomers) between layers of the van der Waals (vdW) material [26, 27, 28], addition of the chirality encoder to the reaction mixture before hydrothermal synthesis [25], or delamination in the presence of the chirality encoder [29]. However, even in these systems, the chirality was relatively weak, and the commonly measured magnitude of the circular dichroism (CD) signal did not exceed several mdeg [25, 26, 27, 28, 29]. It should be noted that despite the absence of clear evidence for a relationship between the properties of the chiroptical materials and the degree of spin polarization, these two parameters are generally assumed to be correlated. In other words, materials with lower chiroptical responses are expected to produce a lower degree of spin polarization. Thus, TMDCs with lower chirality may not necessarily provide a high degree of spin polarization, resulting in lower functionality in spin‐controlled electrochemical applications.

Recently, Gun’ko et al. proposed an alternative method for preparing chiral TMDC nanostructures based on the hydrothermal synthesis of MoS_2_ in the presence of a chirality encoder [30, 31]. This route resulted in significantly higher chirality than that reported in previous studies.

In our work, we utilize the methods proposed by Gun’ko and modified them by adding a dopant to incorporate redox‐active centers into the chiral TMDCs. The resulting chiral materials doped with redox‐active centers were subsequently utilized to improve water oxidation kinetics, while the ratio of the produced hydrogen peroxide to oxygen was used as a marker of CISS.

## 2 Results and Discussion

### 2.1 Main Experimental Concept and Sample Optimization

**FIGURE 1:**
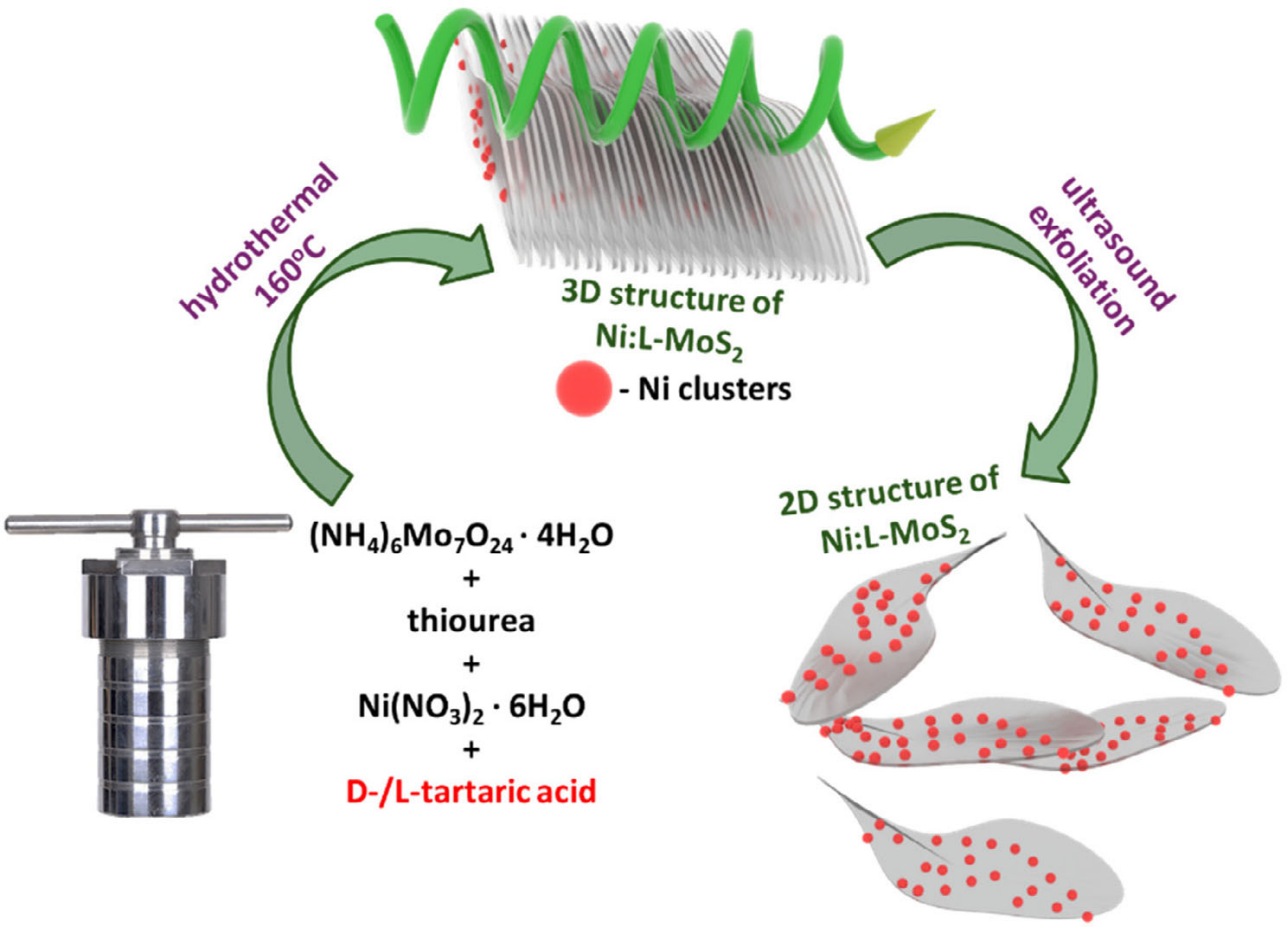
Schematic representation of the experimental approach used for the formation of highly chiral MoS_2_ flakes doped with redox‐active Ni centers.

In our approach, MoS_2_ was synthesized hydrothermally in the presence of a chirality encoder (L‐ or D‐tartaric acid (TA) enantiomers) to obtain highly chiral materials with CD values significantly exceeding those previously reported (Figure 1) [25, 26, 27, 28, 29, 30, 31]. The choice of TA as the chirality encoder was determined through a range of additional experiments using alternative chiral molecules (Figure S1). Unlike the previously reported route [30], we added a nickel (Ni) salt with the expectation that Ni atoms would be incorporated into the structure of chiral MoS_2_ primarily during synthesis. In such a material design, the chirality of the MoS_2_ matrix is responsible for the CISS effect and electron spin polarization, while the Ni atoms ensure high redox activity for water oxidation. After synthesis, the obtained Ni:MoS_2_ powder was subjected to ultrasonic‐assisted delamination, which resulted in the formation of 2D flakes. Subsequently, the flakes were used for water oxidation, and hydrogen peroxide production and oxygen evolution were studied as functions of the chirality and composition of the materials.

Materials with incorporated Ni redox‐active centers were initially intended for high‐activity water oxidation [27, 32]. For this purpose, the optimization of Ni doping was performed first, and the optimization results are presented in the Figures S2–S4. The X‐ray diffrcation (XRD) Characterisation patterns measured for different Ni‐doped samples mainly consisted of characteristic reflections from MoS_2_, without the appearance of reflections from Ni, Ni oxides, or sulfides. The Raman spectra of Ni:MoS_2_ showed the presence of two main modes, E^1^_2g_ and A_1g_, corresponding to the in‐plane and out‐of‐plane lattice vibrations, respectively [33, 34] (Figure S3). Both bands were blueshifted with the addition of Ni, indicating the suppression of lattice vibrations, probably due to dopant‐induced lattice strain. Preliminary characterization of the redox activity of the samples during water oxidation allowed the identification of the optimal Ni concentration (corresponding to the highest catalytic activity among the samples), which was found to be 0.5% (Figure S4). Therefore, this concentration was selected for further experiments (unless otherwise stated).

### 2.2 *Characterization of the Chiral Ni:L‐MoS*_2_ *Structure*

**FIGURE 2:**
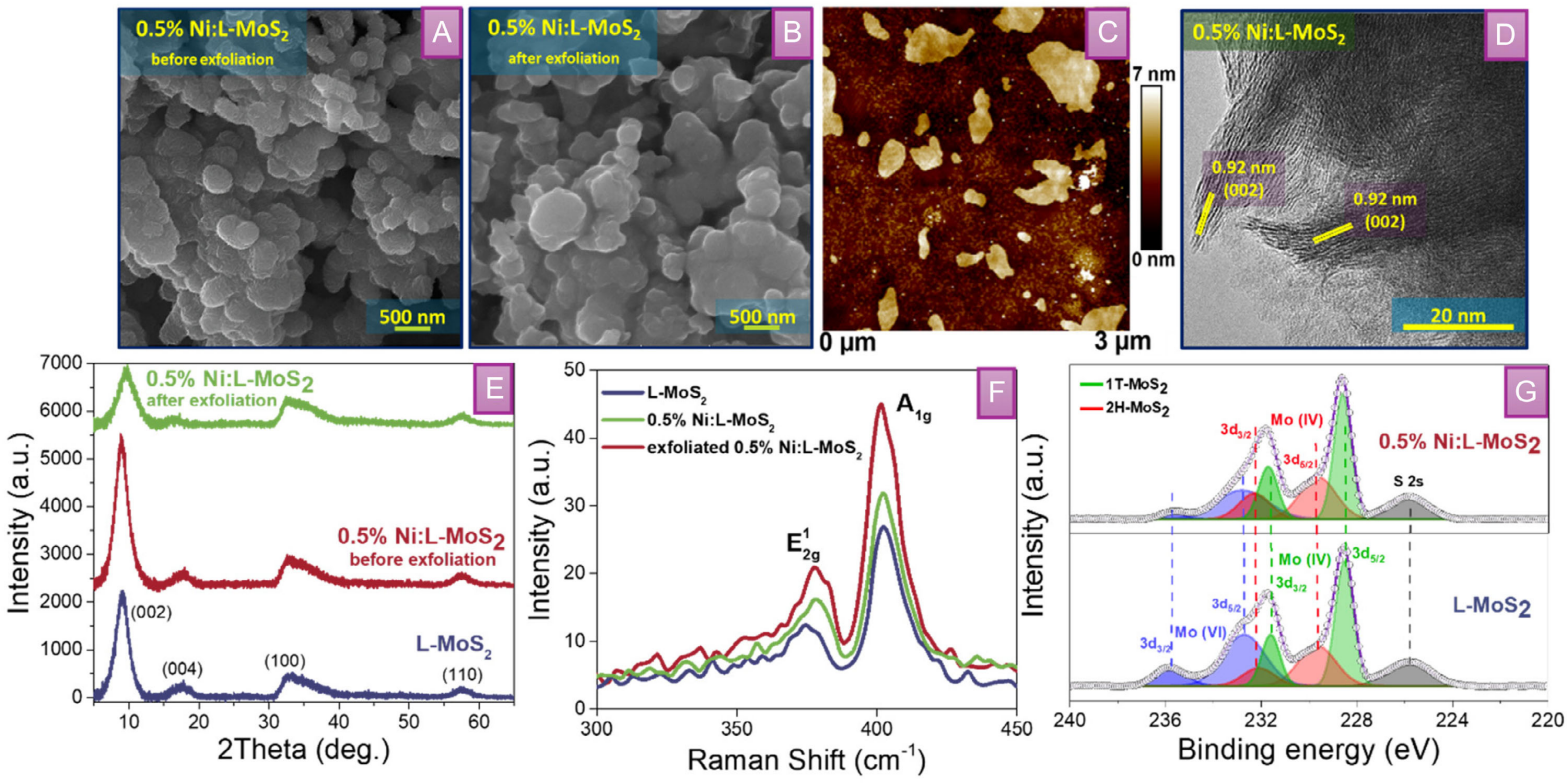
(A,B) SEM images of chiral Ni:L‐MoS_2_ before and after exfoliation; (C) AFM image of particular Ni:L‐MoS_2_ flakes; (D) HRTEM image of Ni‐doped chiral MoS_2_ flake; (E,F) XRD and Raman spectra of chiral Ni:MoS_2_ flakes measured before and after exfoliation; (G) deconvolution of high resolution XPS spectrum of Mo, measured on doped and nondoped chiral MoS_2_ flakes.

In the next step, we proceeded to delaminate Ni:MoS_2_ with the aim of creating a redox‐active 2D material, which was prepared by adding L‐TA and is therefore referred to as Ni:L‐MoS_2_. Scanning electron microscopy (SEM) images of chiral MoS_2_ (with and without Ni doping) captured before and after exfoliation are presented in Figure 2A,B, as well as in Figure S5. Changes in the shape of the material were apparent; the as‐prepared structures with a specific twisted 3D morphology were converted into flatter structures. The Ni doping affected both the structure of the produced 3D material (prior to exfoliation) and the size of the resulting flakes after exfoliation. However, no significant difference in size distribution was observed between the L‐ and D‐MoS_2_ flakes. After drop‐casting a dilute suspension onto a flat silicon surface, atomic force microscopy (AFM) measurements (Figure 2C) revealed the true 2D nature of the resulting Ni:MoS_2_ flakes (additional AFM images are presented in Figure S6 to confirm the 2D nature of the fabricated material). The surface profile measured by AFM indicated that the thickness of the flakes was approximately 4 nm (Figure S7). The transmission electron microscopy (TEM) images (Figures S8 and S9) of the as‐prepared flakes (before delamination) revealed the characteristic layered structure of the material and a homogeneous distribution of Ni atoms in Ni:MoS_2_ across different Ni concentrations. Furthermore, high‐resolution TEM (HRTEM) images of the delaminated 2D flakes revealed an apparent lattice spacing of 0.92 nm, which is typical for the 1T MoS_2_ phase (Figure 2D).

The presence of the 1T phase was also evident from the XRD patterns and Raman spectra of Ni:MoS_2_ (Figure 2E,F), which were measured before and after exfoliation. Compared to the ICSD 254 956 pattern of the 2H phase, 1T‐MoS_2_ exhibits features including a broadened (002) reflection at approximately 9° (indicating increased interlayer spacing), a second‐order reflection at approximately 17°, and additional (100) and (110) reflections that deviate from the positions expected for the 2H structure [35, 36, 37]. These features have also been experimentally observed in 1T‐MoS_2_ produced via hydrothermal synthesis [38, 39]. However, the relatively large widths of the reflections, which are typical for materials obtained by the hydrothermal method, did not allow us to exclude the presence of a more stable 2H phase. A similar pattern was observed for chiral MoS_2_ without the addition of Ni. This suggests that the spacing between the layers is determined more by the intercalation of ammonium ions than by the incorporation of Ni atoms (Figures 2E and S10). MoS_2_ exfoliation did not result in significant changes in the XRD patterns, and only a slight increase in the intensity of the (002) reflection was observed. Raman spectroscopy measurements indicated an apparent shift of the A_1g_ mode after delamination, corresponding to the weakening of the vdW interactions owing to the decrease in the material thickness (Figure 2F). However, we observed an unexpected redshift in the E^1^_2g_ mode [38, 40], which disagrees with published results; nevertheless, a similar shift has also been reported for exfoliated MoS_2_ [41]. Similar to the previous case, a similar Raman peak position was observed for both chiral and achiral MoS_2_ (Figures 2F and S11), confirming that the phonon modes, which are determined by the material composition and crystallinity, were not affected by chirality induction.

The X‐ray photoelectron spectroscopy (XPS) characterization of the chiral flakes, both with and without Ni doping, is presented in Figures 2G and S12–S15. As expected, the main XPS peaks corresponded to molybdenum and sulfur (Figure S12). The Mo 3d and S 2s peaks are clearly visible within the 222–240 eV range. The deconvoluted Mo spectra of both the doped and nondoped chiral MoS_2_ have the expected shape, with noticeably separated Mo 3d_5/2_ and Mo 3d_3/2_ peaks, which is consistent with literature reports (the deconvolution indicates the presence of both the 1T and 2H phases of MoS_2_). The sample also contains molybdenum oxide forms (Mo (IV) and Mo (VI)), which are typically created during hydrothermal synthesis. When MoS_2_ was doped with Ni, Mo (IV) 3d peaks were observed at the same positions, confirming that materials of comparable quality were obtained. However, lower‐intensity oxidation peaks (corresponding to Mo (VI)) were observed in this case, which might suggest that the stabilization of MoS_2_ was induced by nickel doping. The S 2p spectra (Figure S13) show the typical S 2p_3/2_ and S 2p_1/2_ peaks at 161.4 and 162.6 eV, respectively. When Ni atoms were incorporated, the S 2p peaks shifted slightly toward higher energies because of the higher electronegativity of nickel than that of molybdenum. This suggests that the Ni atoms form bonds with S, confirming that Ni is incorporated into the MoS_2_ lattice by substituting for Mo atoms.

### 2.3 Optical Properties and Chirality of Ni:L‐MoS_2_ Flakes

**FIGURE 3:**
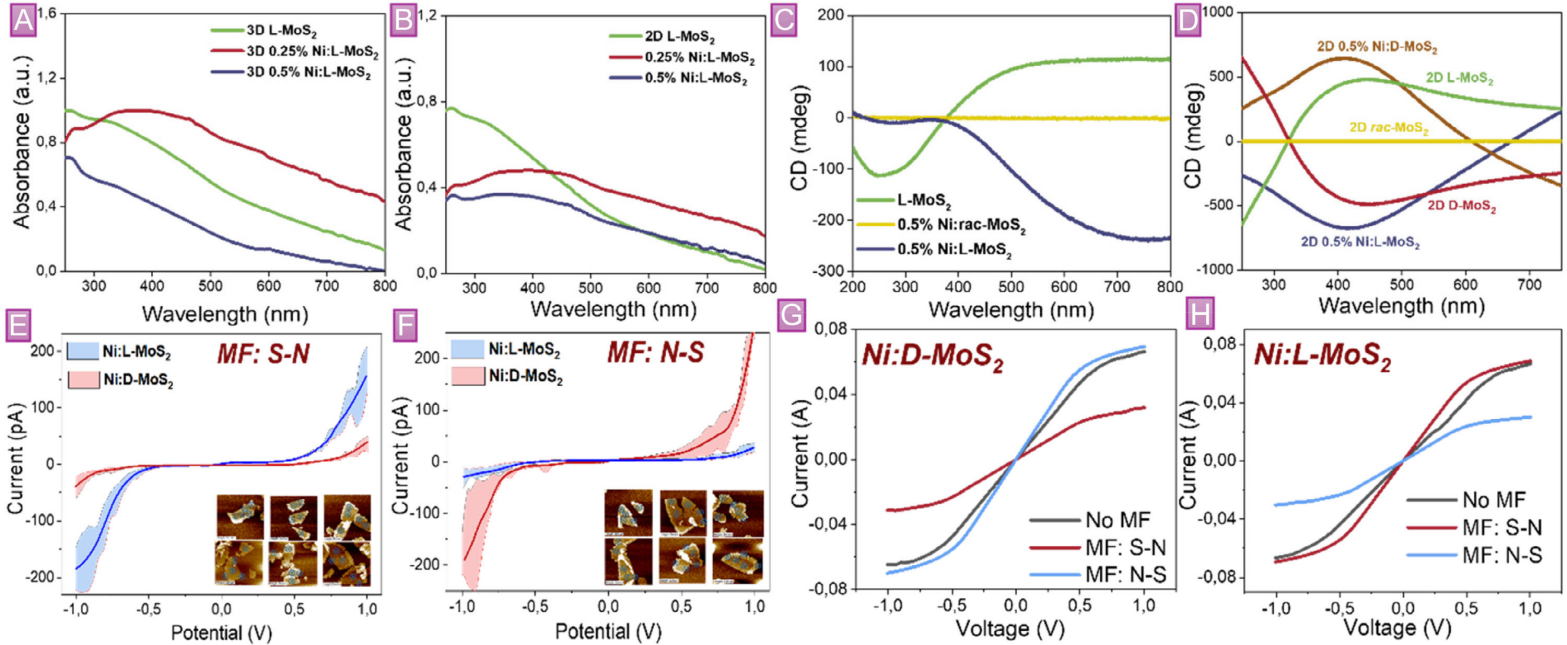
(A,B) UV–vis spectra of L‐MoS_2_ with different Ni doping measured before and after exfoliation; (C) CD spectra of exfoliated L‐MoS_2_ and D‐MoS_2_ flakes with and without Ni doping; the spectrum of MoS_2_ prepared in the presence of racemic TA is provided as a control; (D) measurements of CD response of chiral and achiral MoS_2_ and Ni:MoS_2_ flakes after their immobilization on transparent substrate (solid‐state CD measurements); (E,F) conductive mAFM spectroscopy, performed using preliminary magnetized tips on Ni:L‐MoS_2_ and Ni:D‐MoS_2_ flakes (presented curves are given as an average from 30 measurements in each case, the statistical deviation between separated is highlighted by color areas); (G,H) Mott polarimetry characterization of the chiral flakes with additionally deposited/magnetized Au/Ni layers on the layer of closely packed chiral flakes (*n* = 5 for each presented curve).

In the next step, we proceeded with the optical characterization of the prepared materials. The UV–vis absorption spectra of the differently doped MoS_2_ flakes are shown in Figure 3A,B. The spectra of both 3D and 2D chiral MoS_2_ are dominated by two main electron transitions: the absorption band located at UV wavelengths, which can be attributed to interband electron transitions, and a broad absorption peak appearing at visible wavelengths due to quasi‐plasmon electron oscillations in the partially filled band at the Fermi level, characteristic of the metallic 1T phase of MoS_2_ [38, 42]. The addition of Ni atoms apparently affects the absorption spectrum by resulting in a more pronounced plasmon‐related band, which remains broad but gradually shifts to longer wavelengths as the Ni concentration increases.

The CD spectra showed a significant signal covering the entire visible spectrum for all the samples (Figure 3C,D). For the pristine L‐MoS_2_ (measured in suspension), a strong positive CD band was observed above 400 nm, where a strong negative signal was evident at wavelengths below 300 nm. The D‐MoS_2_ flakes produced mirror‐image CD signals, confirming the formation of a true chiroptical material. In turn, the addition of Ni resulted in a significant change, including changes in the positions of the intersection points, in the CS signal. It should be emphasized that after Ni doping, the CD signal remained very strong, where L‐ and D‐MoS_2_ showed opposite mirror‐image CD signals. In particular, doped MoS_2_ particles prepared using the opposite enantiomer produced an inverse CD signal, as in the case of pristine MoS_2_. It should also be noted that the observed chiroptical activity significantly surpasses that reported in previous studies on MoS_2_ [25, 26, 27, 28, 29], except for the recent works by Gun’ko, which inspired this study [30, 31]. From the UV–vis and CD spectra, the g‐factors were calculated to be 0.354 and 0.232 for 2D L‐MoS_2_ and 2D Ni:L‐MoS_2_, respectively (both at 420 nm). Finally, MoS_2_ flakes prepared with the addition of an enantiomeric racemic mixture (1:1 mixture of L‐ and D‐TA) showed no detectable CD signal, confirming the key role of the enantiopure chirality encoder in the production of chiroptical materials.

The spin polarization arising from material chirality was further confirmed using conductive magnetic (mAFM) measurements, performed with tips that were preliminarily magnetized in opposite directions. The apparent symmetric interplay between the chirality of the Ni:MoS_2_ flakes and the magnetization of the tip is evident from the statistical distribution of the I–V curves and the corresponding average curves. This indicates a common phenomenon arising from the CISS effect: the electrons that pass through the magnetic tip are spin‐polarized, and their probability of passing across the chiral flake surface (or being received by the flakes) depends on the spin alignment (Figure 3E,F). The utilization of conventional Mott polarimetry, performed at a macro scale by adding a Ni layer to the flake surface, also resulted in similar observations, where an apparent dependency of the resistivity of the structure on the magnetization of the Ni layer and the chirality of the flakes was observed (Figure 3G,H). Control measurements performed with nonchiral flakes indicated the absence of I–V behavior related to the preliminary magnetization of the charge receiver or collector layers (Figures S16 and S17).

The conservation of chirality following flake delamination and the formation of a true 2D structure (Figures 2C and S6) also allowed us to speculate on the origin of chirality. Because we did not observe the presence of TA enantiomers in the structure of chiral MoS_2_ (Figure S18), it can be assumed that the chirality originates either from a twisted morphology of the MoS_2_ particles or from the presence of defects in the crystalline lattice of MoS_2_ generated during material preparation. Finally, chirality measurements were performed in the solid state after the flake deposition on a transparent quartz substrate. The obtained results are shown in Figures 3D and S19 confirm that the optical chirality persisted even after the flakes transitioned from a suspension to a solid‐state film form. In other words, the thin films of Ni:L‐MoS_2_ remain strongly chiral and therefore represent suitable platforms for investigating the CISS phenomenon.

### 2.4 Water Oxidation Experiments and Spin Selectivity

**FIGURE 4:**
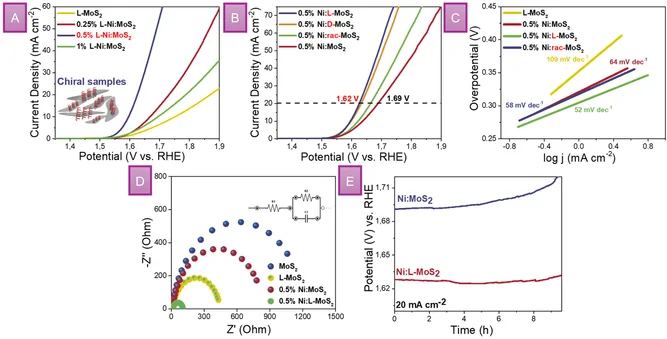
(A) Electrochemical activity of chiral MoS_2_ as a function of Ni doping, measured in the LSV mode; (B) comparison of redox activity in the water oxidation of chiral and achiral (prepared without TA or using TA racemate) Ni‐doped MoS_2_ flakes, performed in the LSV mode; (C) Tafel slopes, calculated from LSV curves for chiral and achiral, pristine and doped MoS_2_ flakes; (D) EIS characterization of pristine and Ni‐doped chiral and achiral MoS_2_ flakes; (E) stability tests, performed using chiral and achiral Ni‐doped MoS_2_ flakes in chronopotentiometry at 20 mA cm^−2^ current density.

Next, the chiral and redox‐active materials were prepared for water oxidation. The main aim was to suppress the formation of undesirable hydrogen peroxide and promote the oxygen evolution reaction. First, we studied the electrochemical activity of the samples as a function of the Ni doping (Figure 4A). The obtained linear sweep voltammetry (LSV) curves show that Ni doping increases the oxygen reduction reaction (OER) activity of MoS_2_. In particular, the incorporation of 0.25% Ni decreased the offset potential from 1.87 V versus reversible hydrogen electrode (RHE) for pristine MoS_2_ to 1.73 V for the doped material. Increasing the Ni concentration to 0.5% resulted in an additional decrease in the offset potential to 1.62 V; however, further increasing the amount of Ni (1% doping) worsened the catalytic activity of the material.

Figure 4B shows a comparison of the redox activities of chiral and nonchiral (prepared using a TA racemate) Ni‐doped samples (0.5% Ni doping was chosen based on previous measurements). The introduction of chirality enhanced the redox activity Ni:MoS_2_, as evidenced by the stronger increase in current density with increasing potential during the LSV scan. Better activity was observed for both L‐ and D‐ chiral MoS_2_ flakes doped with Ni, which apparently overperformed the nonchiral flakes prepared without a TA addition or with the addition of TA racemate. In particular, a shift in the offset potential of the reaction from 1.69 to 1.62 V (at 20 mA cm^−2^) was observed (this result was confirmed by a series of statistically relevant measurements; Figure S20). The calculated Tafel slopes were 109 mV dec^−1^ for chiral MoS_2_, 64 mV dec^−1^ for Ni‐doped nonchiral and racemic Ni:MoS_2_, and 52 mV dec^−1^ for Ni:L‐MoS_2_ (Figure 4C). Therefore, the introduction of chirality significantly accelerates the reaction kinetics for both MoS_2_ and Ni:MoS_2_ flakes, and this enhancement of the water oxidation kinetics can be attributed to the reaction proceeding through alternative pathways with a lower thermodynamic barrier, which is facilitated by spin alignment.

The results of the EIS measurements presented in Figure 4D also confirm the enhanced redox activity of chiral Ni:MoS_2_ compared with that of its achiral counterpart. This was evident from the apparent decrease in the charge–transfer resistance between the electrode and electrolyte (the characteristic semicircles exhibited the smallest radius for chiral MoS_2_ doped with 0.5% Ni). Similar results were obtained when considering the mass and surface activities of the samples: Ni doping and the introduction of chirality significantly enhanced both performance metrics in water oxidation. The mass activity of the samples increased from 61  to 111 Ag^−1^, while the specific activity increased from 0.53 to 1.04 following the introduction of chirality into the Ni:MoS_2_ flakes (Table S1, Figure S21). Finally, stability tests performed in chronopotentiometry mode indicated significantly better stability for chiral Ni‐doped MoS_2_, for which no potential changes were observed while maintaining a constant current density (this can be correlated with spin alignment and reaction‐selectivity tuning, particularly the suppression of hydrogen peroxide production and the prevention of catalyst degradation). Additional material characterization was performed after the stability tests (Figures S22–S24), which indicated the preservation of the crystallinity and shape of the flakes, accompanied by surface oxidation, which does not adversely affect the redox activity (at least for the chiral, spin‐selective material).

Therefore, the results of the electrochemical characterization of the samples clearly indicate an increase in the redox activity of the samples during water oxidation. This reaction proceeds through multi‐electron transfer via several pathways, leading to the formation of singlet or triplet oxygen and hydrogen peroxide. The CISS effect suppresses the formation of hydrogen peroxide and promotes the formation of otherwise forbidden triplet oxygen. The formation of this oxygen species proceeds through a lower thermodynamic barrier, which explains the enhanced reaction kinetics. To monitor the reaction and the impact of the CISS effect, we further investigated the kinetics of oxygen production and the oxygen‐to‐peroxide ratio using two approaches: photo‐colorimetry (Figure S25) for determining the H_2_O_2_ concentration and differential electrochemical mass spectrometry (DEMS) measurements of oxygen as the main reaction product (Figure S26).

**FIGURE 5:**
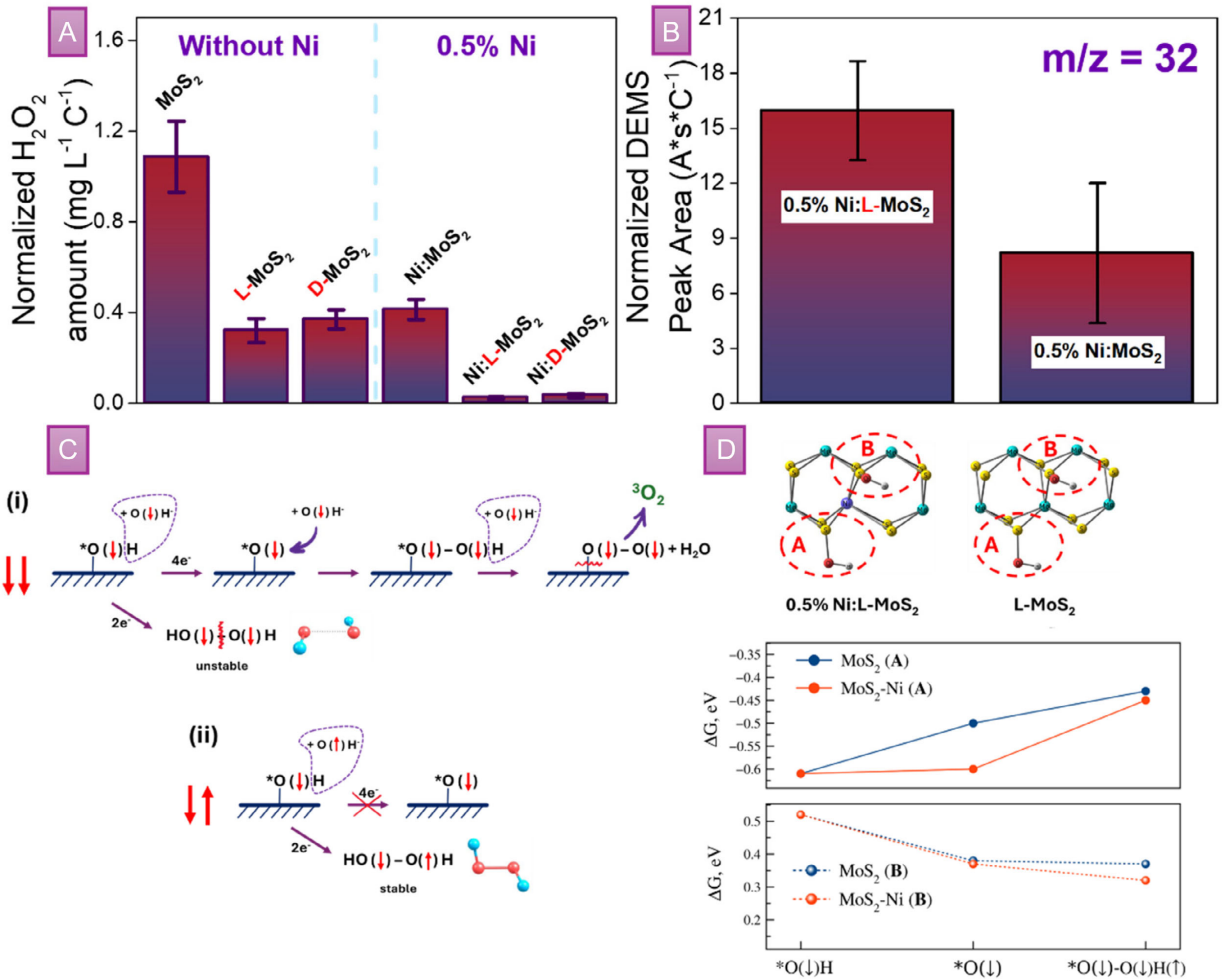
(A) Normalized on passed charge H_2_O_2_ production rate obtained on chiral and achiral MoS_2_ flakes with or without Ni doping; (B) DEMS measured oxygen evolution on chiral and achiral flakes, presented as characteristic O_2_ peak surface area normalized on the overall charge passed through the system; (C) illustration of the considered 2e^−^ and 4e^−^ reduction pathway of OER for (i) spin‐parallel and (ii) spin‐antiparallel hydroxyl arrangements; (D) free energy profiles for OER at the different active sites A and B for nickel‐doped and pure MoS_2_ fragments. (A and B), the error bars represent the mean ± SD (*n* = 3).

The results of the H_2_O_2_ production tests are presented in Figure 5A as a function of Ni doping and the introduction of chirality. To avoid misleading results related to differences in reaction kinetics, we used the H_2_O_2_ production rate based on the amount of electrical charge consumed in the electrochemical cell. In the case of pristine achiral MoS_2_, both oxygen and hydrogen peroxide were produced (as confirmed by DEMS measurements). The addition of Ni led to a significant change in the reaction efficiency, evident from the decrease in the relative amounts of H_2_O_2_ produced and the increase in its absolute amount due to the enhanced reaction kinetics (Figure S27). Therefore, even the addition of Ni can tune the reaction toward the desired oxygen‐evolution pathway. However, even more promising results were obtained with the introduction of chirality and the related CISS effect. In this case, even pristine MoS_2_ showed a significant suppression of hydrogen peroxide production. In turn, a more pronounced suppression was observed for Ni‐doped chiral MoS_2_. Only minute amounts of hydrogen peroxide were detected, indicating that the transferred charge carriers were mainly consumed for oxygen evolution.

The relative amounts of the evolved oxygen were determined using DEMS experiments performed in a time‐resolved regime under a constant electric current. First, a characteristic oxygen‐related peak was observed immediately after the external power source was switched on (Figure S28). The use of chiral and achiral Ni‐doped MoS_2_ resulted in different peak areas, reflecting the amount of oxygen produced. In particular, a higher peak area (normalized to the total charge passed through the system) for chiral Ni:MoS_2_ was evident (Figures 5B and S29). This confirms that the reaction shifted toward oxygen evolution owing to the CISS effect, whereas the side reaction leading to hydrogen peroxide formation (which also consumes charge carriers) was significantly suppressed.

### 2.5 Density Functional Theory (DFT)‐Based Estimation of CISS Impact on Water Oxidation

To explain the impact of the CISS effect on water oxidation kinetics and selectivity, we performed a series of DFT calculations to reveal the specific role of spin selectivity and Ni‐doping ions in the formation of triplet or singlet oxygen and H_2_O_2_ production (see Figure 5C,D). We experimentally observed the appearance of the CISS effect owing to the introduction of chirality in the materials (i.e., twisting of the flake shape); however, common DFT calculations are unable to reproduce the characteristic scales of materials exhibiting the required radius of curvature. Therefore, we investigated scenarios in which spin separation of hydroxyls occurred near the flake surface and examined various pathways for the formation of peroxide and triplet oxygen. Additionally, we focused our DFT calculations on key OER steps where a fundamental difference between the doped and pure materials was observed as a function of the intermediate spin polarization (the remaining steps did not provide any additional information). Plane‐wave DFT calculations were performed to elucidate the effect of Ni doping on the OER of the MoS_2_ flakes under CISS conditions (details are provided in the Supporting Information). The proposed model considers the physical separation of spin‐oriented hydroxyl ions in local proximity to the chiral flakes. This led to the accumulation of spin‐parallel hydroxyls near different surfaces, which then proceeded to the OER (Figure 5C). Without the CISS effect, the spin‐antiparallel hydroxyls reached approximately equal concentrations near the OER sites. Following Viswanathan et al. [43] regarding the selectivity of metal surfaces for the 2e^−^ and 4e^−^ oxygen reduction pathways, we calculated the free energy profiles of the OER. For comparison, we considered nickel‐doped (Ni:MoS_2_) and pure (MoS_2_) materials (Figure 5D). A fragment of the MoS_2_ monolayer (100) with a single nickel atom served as the OER reaction site on the front side (A) and upper side (B), as shown in Figure S30. It has been previously reported [44] that doping MoS_2_ with Ni facilitates the kinetics of the hydrogen evolution reaction under alkaline conditions by reducing the energy barrier for water dissociation and OH^−^ desorption. The presence of unsaturated S atoms favors proton adsorption, accelerating the hydroxyl generation.

DFT geometry optimization of the neutral complex of two hydroxyl groups in the triplet state near the flake surface, corresponding to the 2e^−^ reduction pathway for spin‐parallel orientation (Figure 5C(i)), led to dissociation and prevented peroxide formation. This instability of the H_2_O_2_ complex in the triplet state was previously reported in [45]. At the same time, spin‐antiparallel complex optimization resulted in a stable singlet ground‐state structure of H_2_O_2_, suggesting that this is the main channel of peroxide formation (Figure 5C(ii)). The reaction path with a total gain of 4e^−^ for spin‐antiparallel complexes is practically forbidden (Figure 5C(ii)). Therefore, triplet oxygen (^3^O_2_) formation is associated with several stages of chemical transformation along the 4e^−^ reduction pathway for spin‐parallel complexes (Figure 5C(i)). In our model, this reaction channel best explains the experimentally observed increase in electric current and redox activity as a result of the CISS effect.

The free energy profiles corresponding to the OER at the upper site B revealed a mostly positive free‐energy change, practically eliminating this site from the reaction (Figure 5D). In contrast, the free energy profiles corresponding to the OER at front site A clearly show a preference for the reduction process, whereas in the case of a nickel‐doped fragment, the reaction proceeds with even lower energy requirement. The dehydrogenation step between the *OH and *O intermediates is more favorable, requiring only 0.1 eV for the nickel‐doped fragment. This explains why the amount of peroxide formed in the Ni‐doped flakes was lower than that in the pure flakes under CISS conditions. This decrease results from the increased rate at which the hydroxyl groups transform into triplet oxygen. It is important to note that peroxide formation still occurs even when spin‐antiparallel hydroxyls are effectively separated. Incomplete spin polarization or long‐range effects away from the flakes may occur when spin‐antiparallel hydroxyls interact to form complexes.

## 3 Conclusion

In this study, we demonstrated the fabrication of two‐dimensional, highly chiral MoS_2_ flakes with incorporated Ni atoms, inspired by the work of Gun’ko [30, 31], for use in water oxidation under CISS conditions. Chiral flakes were produced by hydrothermal synthesis in the presence of a chirality encoder (organic TA enantiomers). Ni doping enabled the combination of previously reported flake chirality and redox activity, while also affecting the sign and shape of the CD signal. Following delamination, the flakes were employed in water oxidation reactions, and the efficiency and selectivity of these reactions were monitored in relation to the presence of Ni dopants and the induction of chirality. The incorporation of Ni atoms significantly increased the redox activity of the materials. However, an even more significant enhancement in the kinetics of water oxidation was observed upon the introduction of chirality, and the best results were obtained using chiral and Ni‐doped MoS_2_ flakes. In turn, the amount of hydrogen peroxide, as determined by photocolorimetric measurements, was significantly suppressed by the CISS effect, whereas the amount of oxygen increased, as revealed by DEMS measurements. Furthermore, quantum‐mechanical calculations provided insight into the impact of the CISS phenomenon on reaction efficiency and selectivity by considering the reaction pathway involving the formation of triplet oxygen.

## 4 Experimental Section

### 4.1 Materials

Ammonium molybdate tetrahydrate (BioUltra, ≥99.0%), thiourea (ACS reagent, ≥99.0%), L‐(+)‐TA (ACS reagent, ≥99.5%), D‐(−)‐TA (≥99.0%), DL‐TA (≥99.0%), nickel (II) nitrate hexahydrate (99.999% trace metals basis), water (deionized for synthesis) were purchased from Sigma Aldrich.

### 4.2 Samples Preparation

#### 4.2.1 *Synthesis of L‐/D‐/rac‐MoS*_2_ *and Ni:L‐/D‐/rac‐MoS*_2_

The synthesis of MoS_2_‐based structures was carried out according to the procedure published in [30] with some modifications. Briefly, a 0.05 M aqueous solution of L‐/D‐/rac‐TA was prepared, followed by the addition of ammonium molybdate tetrahydrate (0.65 mmol) and thiourea (12 mmol) to the resulting solution. Then, a certain amount of nickel nitrate hexahydrate was added to the solution, which was treated with ultrasound under N_2_ bubbling. The resulting solution was then placed in a Teflon‐lined steel autoclave and heated in an oven at 160 °C for 18 h. The resulting solid precipitate was thoroughly washed with distilled water and absolute ethanol, dried under vacuum at 45 °C, and used for further experiments.

#### 4.2.2 *Delamination of L‐/D‐/rac‐MoS*_2_ *and Ni:L‐/D‐/rac‐MoS*_2_

The delamination process of the materials was carried out by high‐power ultrasound treatment in a closed flask under an inert atmosphere. In particular, 50 mg of powder was immersed in 40 mL of N‐methylpyrrolidone and exposed to ultrasound (300 W) for 2 h. The obtained suspension was centrifuged (4000 rpm, 7 min) to remove any nondelaminated particles. Then, the resulting suspension was separated by centrifugation (10000 rpm, 45 min), washed several times with ethanol, and dried under vacuum at 70 °C.

### 4.3 Characterisation Techniques

UV–vis absorption spectra were obtained using a Lambda 25 UV/Vis/NIR spectrometer (PerkinElmer, USA). CD spectra were obtained using a J‐810 spectrometer (Jasco, Japan) with a scanning speed of 100 nm·min^−1^, a bandwidth of 1 nm, and the standard sensitivity setting. An integration time was set to 1 s for each spectral point. The *g*‐factor was calculated using the following equation [46]: *g* = Δ*A/A*, where Δ*A* is the CD signal and *A* is the corresponding absorbance.

Raman spectra were collected using a Thermo Scientific Raman dispersive spectrometer (model DXR3), equipped with an Olympus confocal microscope. Measurements were taken with an excitation wavelength of 532 nm, a laser power of 0.1 mW, a measurement time of 30 s, and an average of 10 spectra. XRD diffractograms were recorded using an XRDynamic 500 (Anton Paar) diffractometer for 30 min with Cu Kα radiation (1.5405 Å). XPS data were obtained using an EnviroESCA device (SPECS) in ultra‐high vacuum conditions, using a monochromatic AlK Alpha X‐ray source (1486.71 eV). A pass energy was set at 20 eV, a measurement step was 0.1 eV for high‐resolution spectra and 0.5 eV for survey spectra, and a dwell time was 0.3 s per point. SEM was performed using a Tescan Lyra3 GMU microscope at an acceleration voltage of 20 kV.

Scanning transmission electron microscopy (STEM), including high‐resolution imaging (HRTEM), was performed on a (STEM JEOL 2200FS, Jeol Ltd, Japan) equipped with an FEG cathode operated at an acceleration voltage of 200 kV, with an in‐lens secondary electron detector, and a Centurio Large Angle SDD‐EDX detector. AFM surface topographies were measured using an Icon system (Bruker) in Peakforce mode.

Conductive mAFM experiments were performed using Ni:Co‐coated tips that had been magnetized preliminarily in the opposite direction (nonmagnetized Ni:Co tips were used for control experiments). The Ni:L‐MoS_2_ and Ni:D‐MoS_2_ flakes were deposited from a diluted suspension onto a thin Au layer. First, the sample surface was mapped to reveal the position of the flakes. Then, the tip was randomly placed on the MoS_2_ flake surface and I–V curves were obtained within the −1  to 1 V potential range. These measurements were repeated on separately prepared flakes at several points, and the results are presented as averaged curves, including the deviation between the individual I–V curves (30 I–V curves were obtained for each particular tipmagnetization and MoS_2_ flake type).

Mott polarimetry was performed using the subsequent deposition of Ni and Au layers on the surface of closely packed flakes (approximately 5 µm thick layer on a conductive substrate), without disrupting the vacuum cycle using a Nano 36 deposition system. The Ni layer was then magnetized in an external magnetic field of 0.95 T, and I–V curves were measured in relation to the flake's chirality and Ni magnetization.

#### 4.3.1 Electrochemical Measurements

Water oxidation was studied by LSV, cyclic voltammetry, chronoamperometry, and chronopotentiometry. Electrochemical measurements were conducted in a three‐electrode cell using a PalmSens EmStat 4S potentiostat (Palm Instruments, the Netherlands). The working electrode was a catalyst‐loaded glassy carbon electrode (GCE), the counter electrode was platinum, and the Hg/HgO electrode was used as the reference electrode. Typically, 8 mg of powder ethanol suspension mixed with 5.0 wt% Nafion solution was loaded onto a polished GCE and dried at room temperature. LSV was carried out in an N_2_‐saturated 1 M KOH solution with a scan rate of 10 mV·s^−1^. Chronoamperometry measurements were performed in 1 M KOH at 1.6 V versus RHE. At certain time intervals, the electrolyte was transferred to cleaned polypropylene centrifuge tubes and used for the colorimetric H_2_O_2_ test.

Photometric peroxide measurements were performed using a commercial peroxide test kit purchased from Sigma–Aldrich. The pH value of the electrolyte solution obtained after chronoamperometry experiments was adjusted to 10 by adding a few drops of diluted sulfuric acid.

The stability of the catalyst (chiral and nonchiral 0.5% Ni:MoS_2_ flakes) was recorded at a constant current density of 20 mA·cm^−2^ in 1 M KOH solution for 9 h.

DEMS measurements were performed with a Pfeiffer Vacuum Omnistar GSD 320 device and a PalmSens EmStat 4S potentiostat. The reaction was carried out in a three‐electrode configuration using a H‐cell. Each compartment contained 25 mL of electrolyte to ensure the separation of the counter and working electrodes. Mass spectrometry was performed with an ionization energy of 70 eV, a dwell time of 1 s per mass, and detection via a secondary electron multiplier detector.

All measured potentials were converted to a potential versus the RHE using the following equation: *E*_RHE_ = *E*_Hg/HgO_ + 0.098 + 0.059 * pH*.*

The electrochemically active surface area (ECSA) was calculated using the double‐layer capacitance (*C*_DL_) at the electrolyte/electrode interface using the following equation: ECSA = *C*_DL_/*C*_S_, where *C*_S_ is the specific capacitance. To measure the *C*_DL_, the potential was swept from 0.29 to 0.39 V versus RHE (in the non‐Faradaic potential region) at different scan rates (20, 30, 40, 50, 60 and 70 mV s^−1^). The measured difference in currents (Δ*I*) was plotted as a function of scan rate, and the corresponding *C*_DL_ was calculated by linear fitting of the slopes. *C*_S_ was estimated to be equal to 0.035 mF cm^−2^ according to Henckel et al. [47].

The mass activity (A·g^−1^) was calculated using the equation: mass activity = *j*/*m*, where *j* is the current density (mA·cm^−2^) at *η *= 1.62 V, and *m* is the catalyst loading (g).

The specific surface activity was calculated according to the equation: specific activity = *j*/ECSA, where *j* is the current (mA) at *η *= 1.62 V, and ECSA is the electrochemically active surface area (cm^2^).

### 4.4 DFT Calculations

The DFT calculations were carried out with the CP2K package [48]. The DFT calculations used the combination of the Gaussian and plane‐wave (GPW) [49] scheme for electronic density representation, double‐ζ valence plus polarization (DZVP) basis sets of the MOLOPT [50] type to describe the valence electrons, and norm‐conserving Goedecker–Teter–Hutter (GTH) [51] pseudopotentials to approximate the core electrons. The generalized gradient approximation (GGA) of the Perdew−Burke−Ernzerhof (PBE) [52] functional was used to describe the exchange–correlation interactions. DFT‐D3 method [53] was adopted to consider the vdW interactions. The plane‐wave basis set was truncated at an energy cutoff of 600 Ry. For wave function optimization, we used the orbital transformation (OT) method with the FULL_ALL preconditioner, a small energy gap value (0.005 a.u.), and the DIIS minimizer. The structures were optimized using a self‐consistent field convergence threshold of 1E‐7. For the MoS2 flake (Figure S30) description, we used a bulk structure placed in a 35 × 35 × 15 Å3 size box with periodic boundary conditions in the XY plane.

## Funding

This work was supported by Grantová Agentura České Republiky (23‐05197S) and OP JAK (CZ.02.01.01/00/22_008/0004617).

## Conflicts of Interest

The authors declare no conflicts of interest.

## Supporting information

Supplementary Material

## Data Availability

The data that support the findings of this study are available from the corresponding author upon reasonable request. Some of the data is available at https://doi.org/10.5281/zenodo.21592233.
