# Supplementary material for: Highly Chiral MoS2 Flakes With Redox‐Active Centers for Spin Selective Water Oxidation
Source: Small Sci. 2026 Aug 17;6(8):e70356. doi: 10.1002/smsc.70356 (PMC13489294; doi:10.1002/smsc.70356)
Supplement: Supplementary file 1 [file SMSC-6-e70356-s001.pdf]

Supporting Information for:
Highly Chiral MoS_2 Flakes with Redox-Active Centres for Spin Selective Water Oxidation.

Anastasiia Tulupova[a], Denis Zabelin[a], Ivan Kirishiantsev[a], Ruslan Ramazanov[b], Rashid Valiev[b], Tomas Hrbek[c], Lucia Bajtosova[d], Miroslav Cieslar[d], Alena Michalcova[e], Vladislav Buravets[a], Vasilii Burtsev[a], Marie Urbanova[f], Vaclav Svorcik[a], Oleksiy Lyutakov[a*]

[a] *Department of Solid State Engineering, University of Chemistry and Technology, 16628 Prague, Czech Republic.*

[b] *Department of Chemistry, University of Helsinki, FI-00014 Helsinki, Finland*

[c] *Department of Surface and Plasma Science, Faculty of Mathematics and Physics, Charles University, 180 00 Prague, Czech Republic*

[d] *Faculty of Mathematics and Physics, Charles University, Prague 12116, Czech Republic*

[e] *Department of Metals and Corrosion Engineering, University of Chemistry and Technology, 16628 Prague, Czech Republic#*

[f] *Department of Physics and Measurements, University of Chemistry and Technology, 16628 Prague, Czech Republic*

Table S1 Calculated values of mass activity and surface activity for chiral and non-chiral MoS_2 doped with 0.5 % Ni.

Sample	ECSA (cm^2)	Mass activity (A/g)	Specific activity
0.5% Ni:L-MoS_2	1.34	111.46	1.04
0.5% Ni:MoS_2	1.08	60.57	0.53

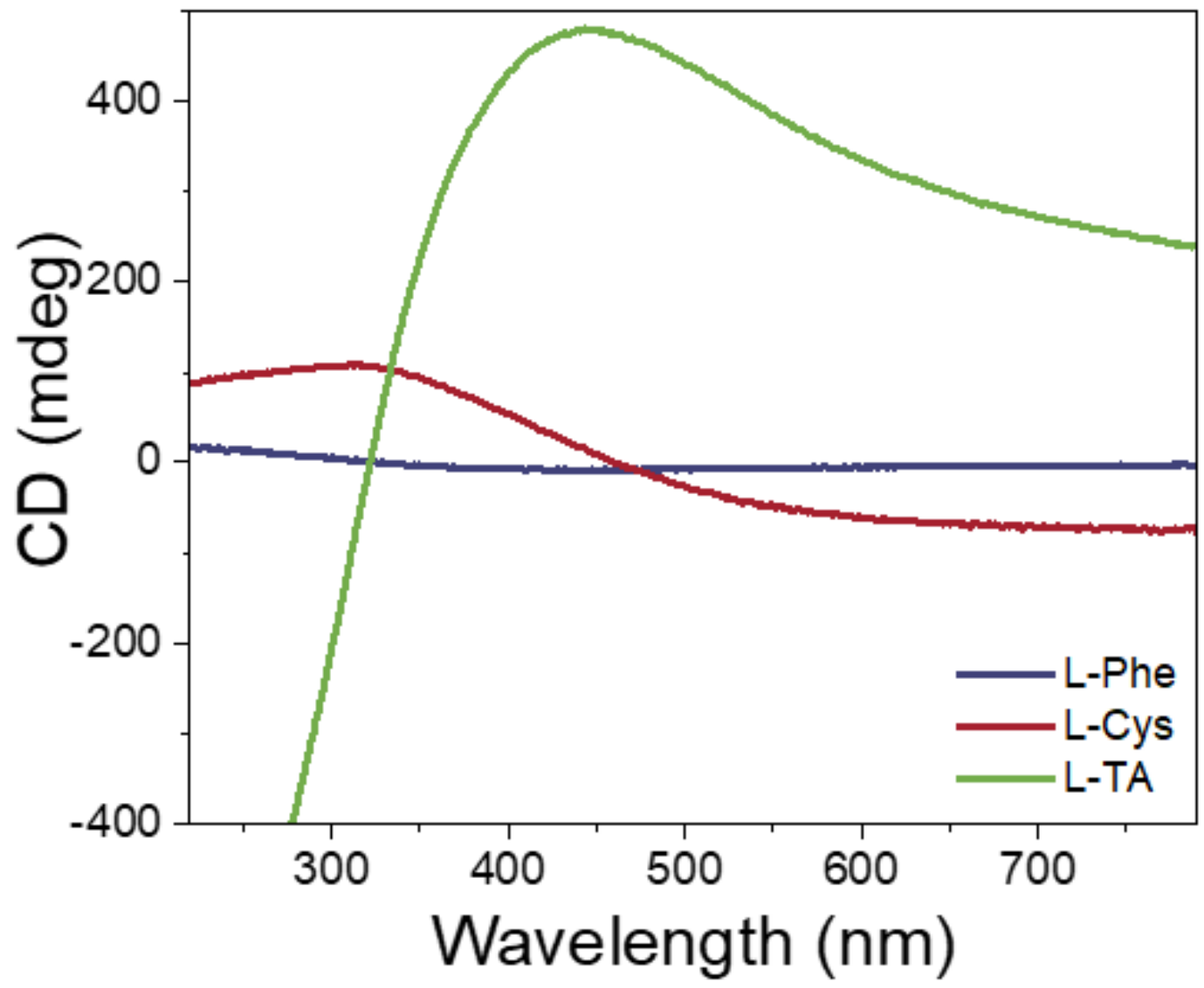

Figure S1. CD spectra of MoS_2 flakes, prepared using the phenylalanine and cysteine L-enantiomers, compared with the flakes prepared using L-TA.

Figure S1 – description note.

During the optimization of chiral MoS_2 synthesis, several chiral encoders were checked. The best results, obtained with different enantio-pure organic molecules (phenylalanine, cysteine and tartaric acid) are presented in Fig. S1. As is evident, the utilization of TA produces better results, which indirectly correlate with Gun'ko works [S1, S2].

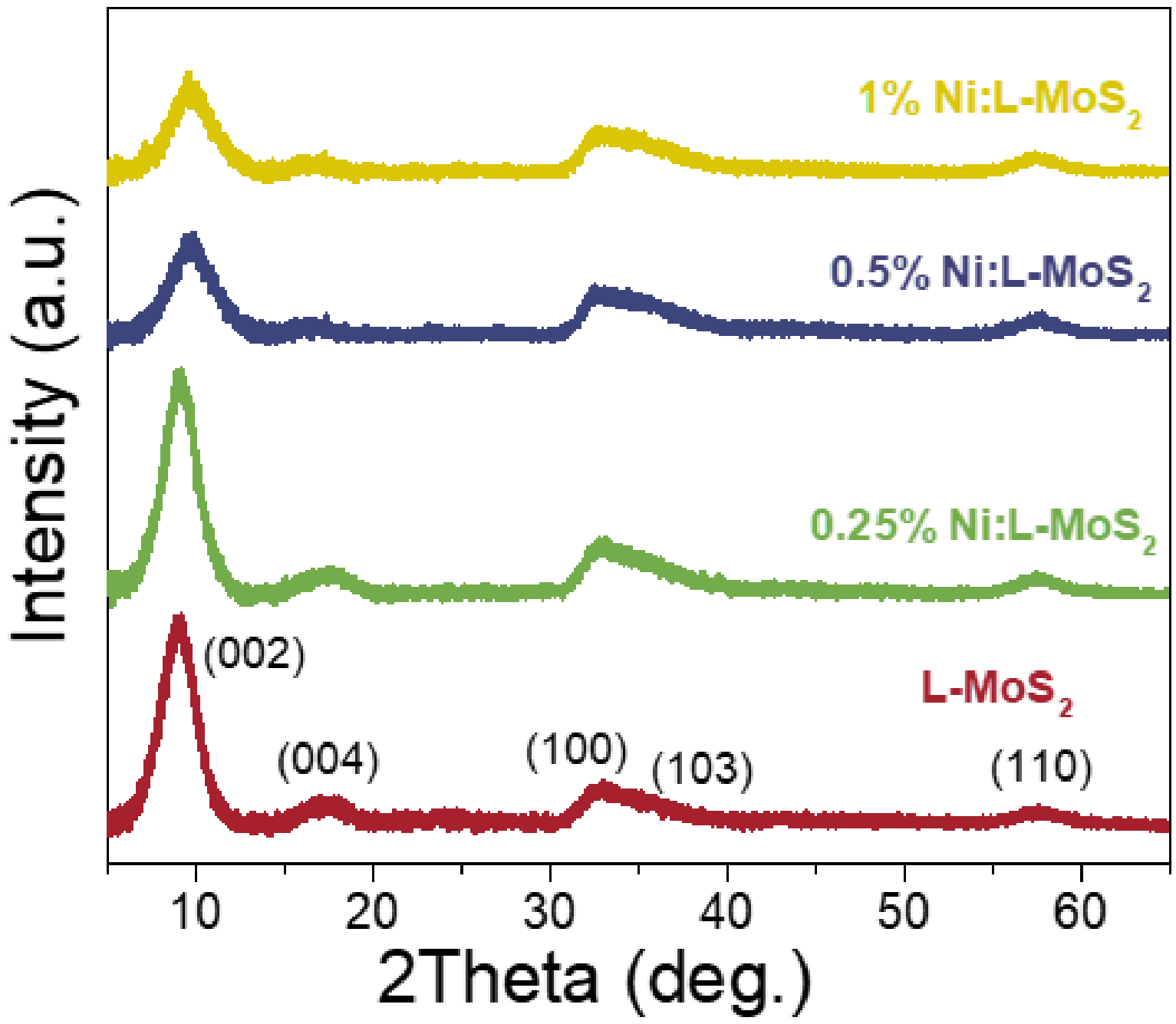

Figure S2. XRD patterns of pristine L-MoS_2 and Ni:L-MoS_2 powder (exfoliated) with the different amounts of incorporated Ni atoms.

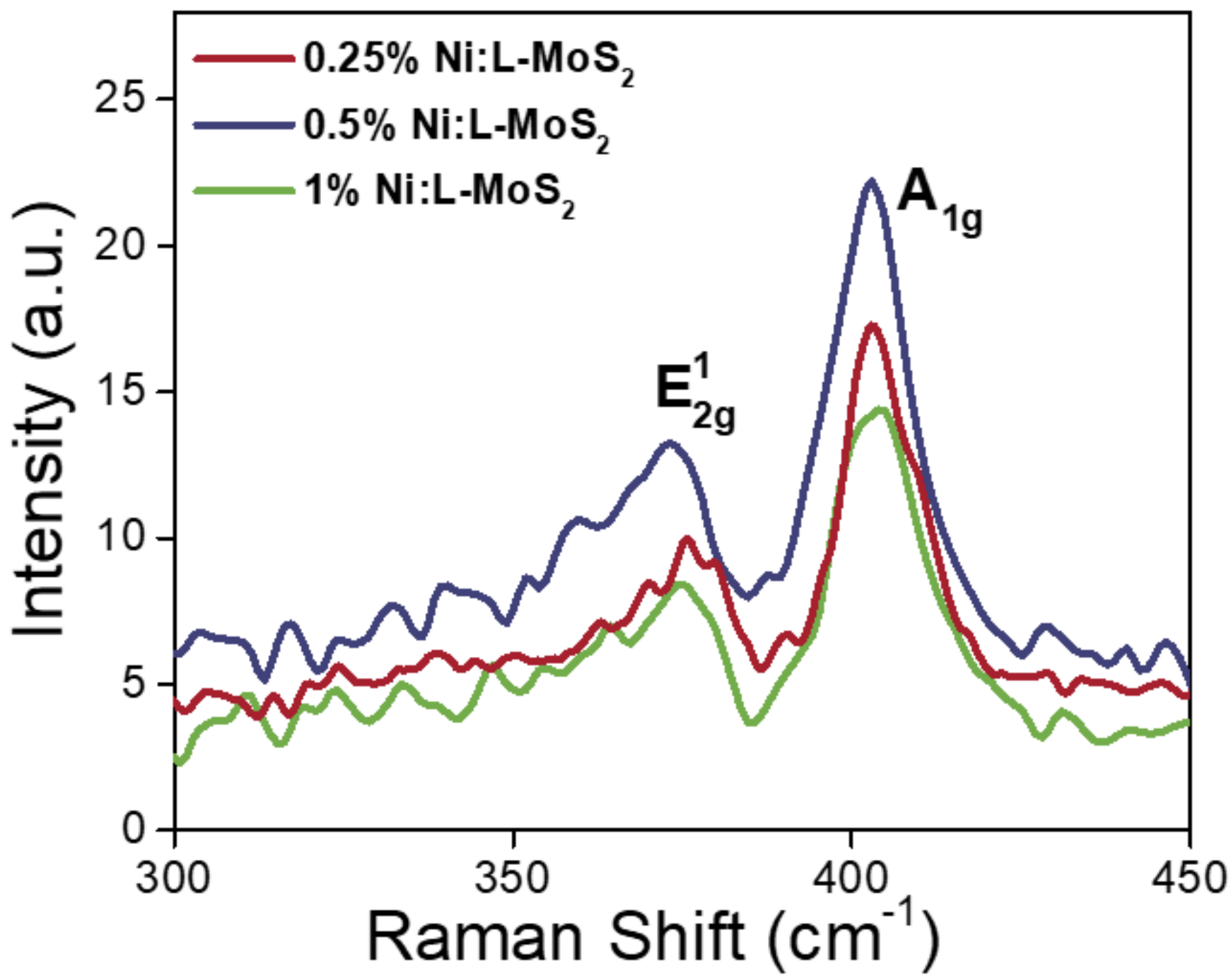

Figure S3. Raman spectra of differently doped chiral MoS_2 flakes.

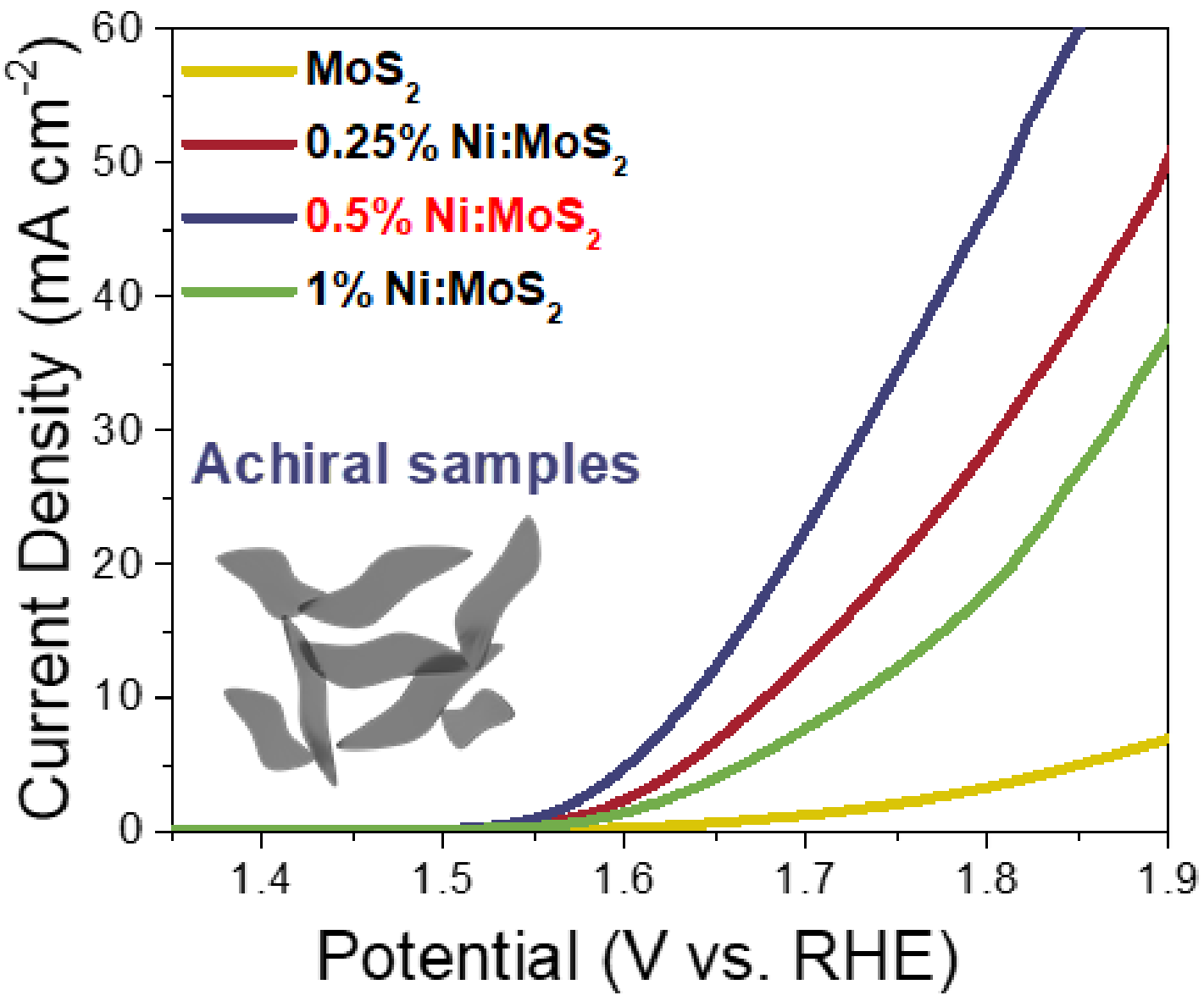

Figure S4. Electrochemical characterisation (LSV mode) of achiral, Ni-doped L-MoS_2 flakes.

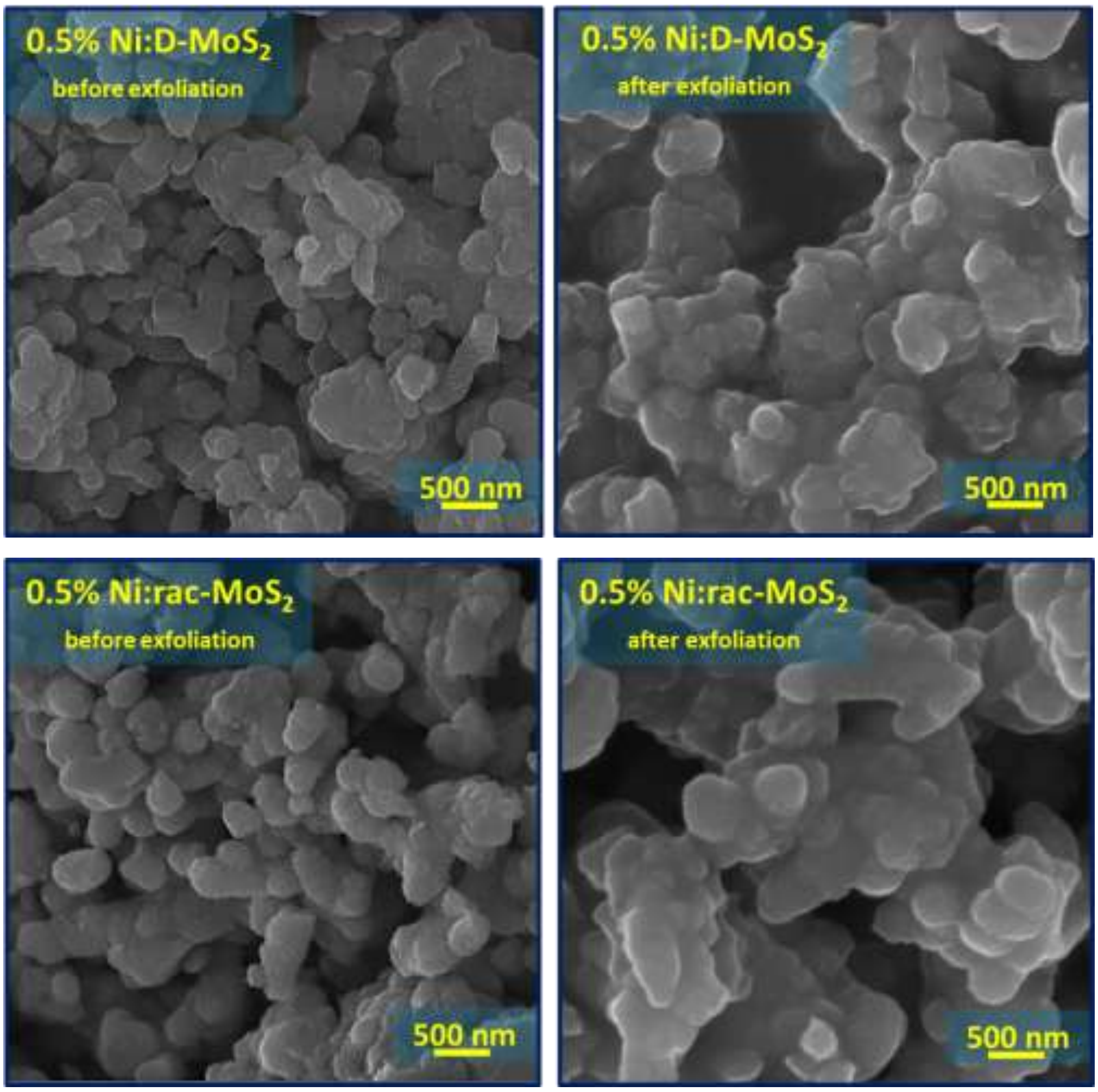

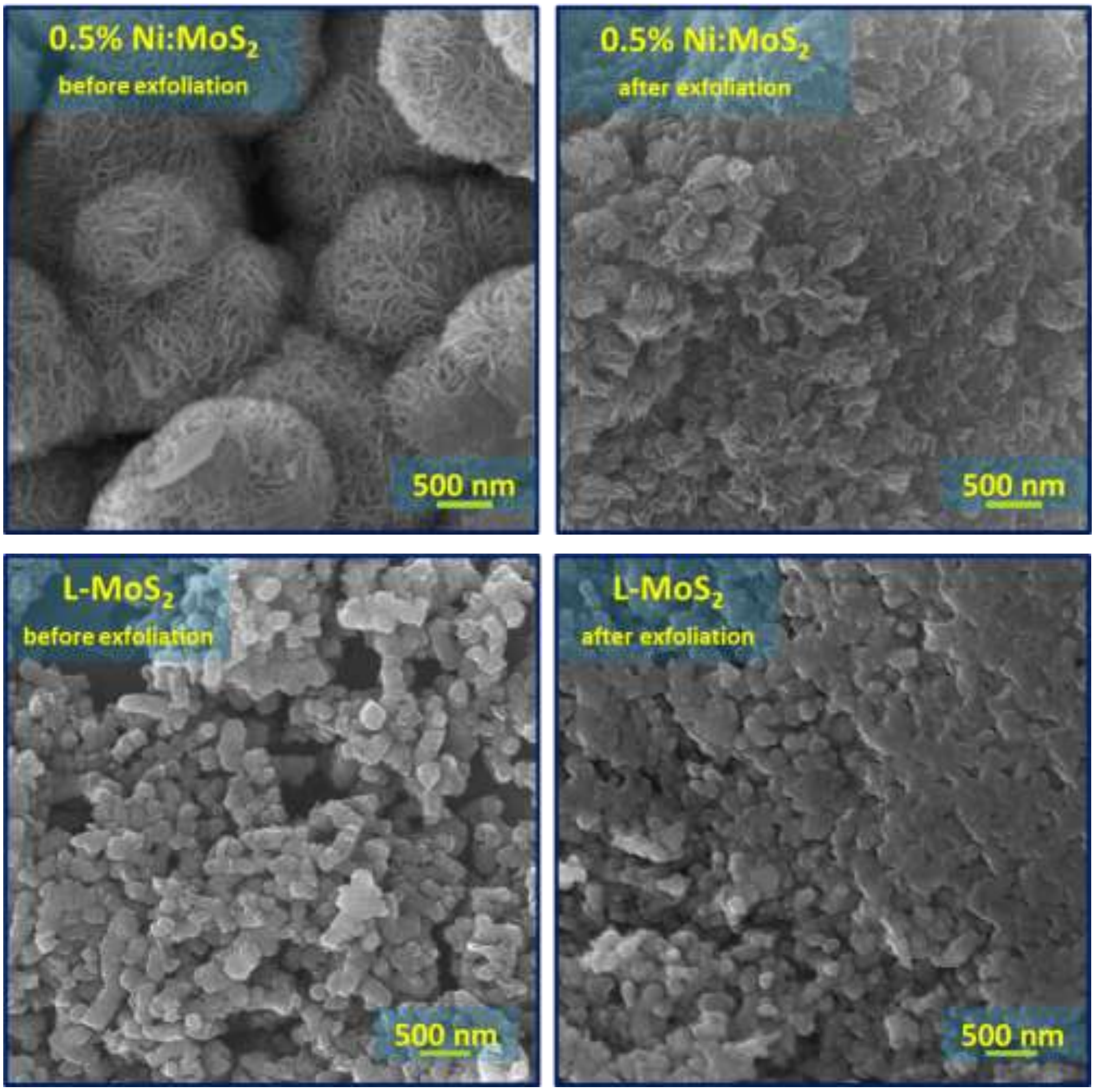

Figure S5. Additional SEM images of pristine and Ni-doped MoS_2 flakes (chiral or achiral) measured before and after exfoliation.

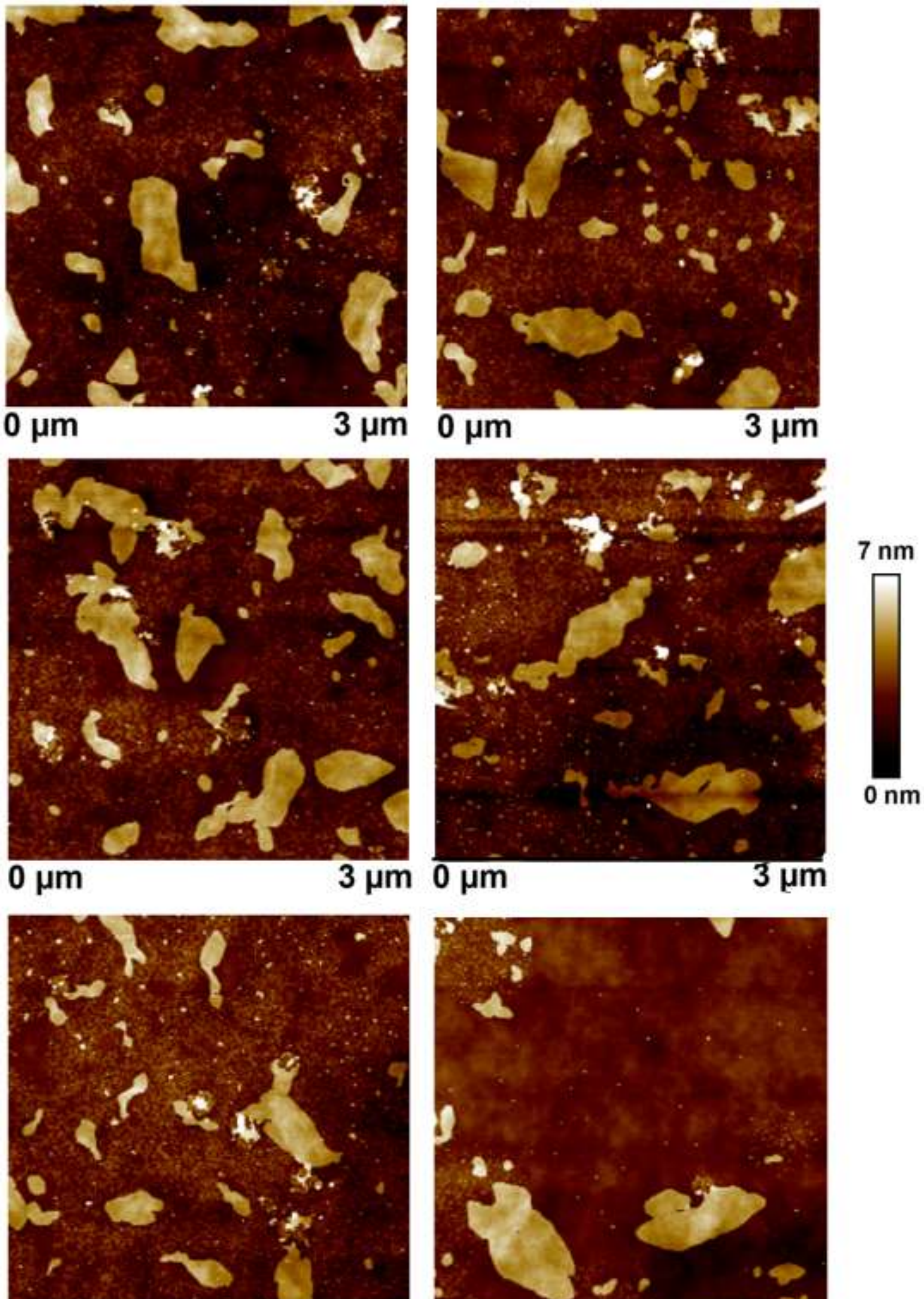

Figure S6. Additional AFM images of chiral, exfoliated flakes (0.5% Ni:L-MoS_2).

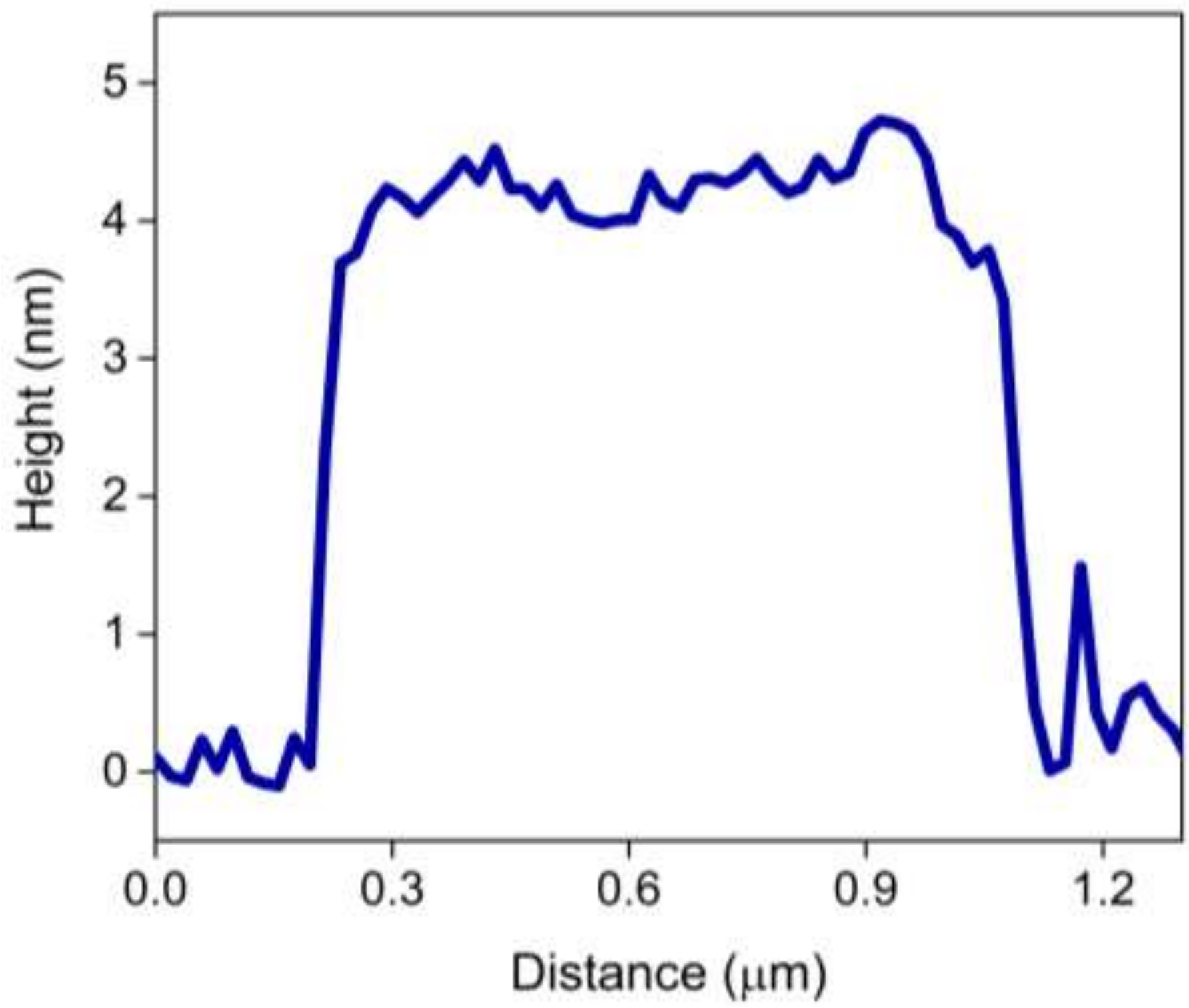

Figure S7. AFM-measured surface profile of single 0.5% Ni:L-MoS_2 flake.

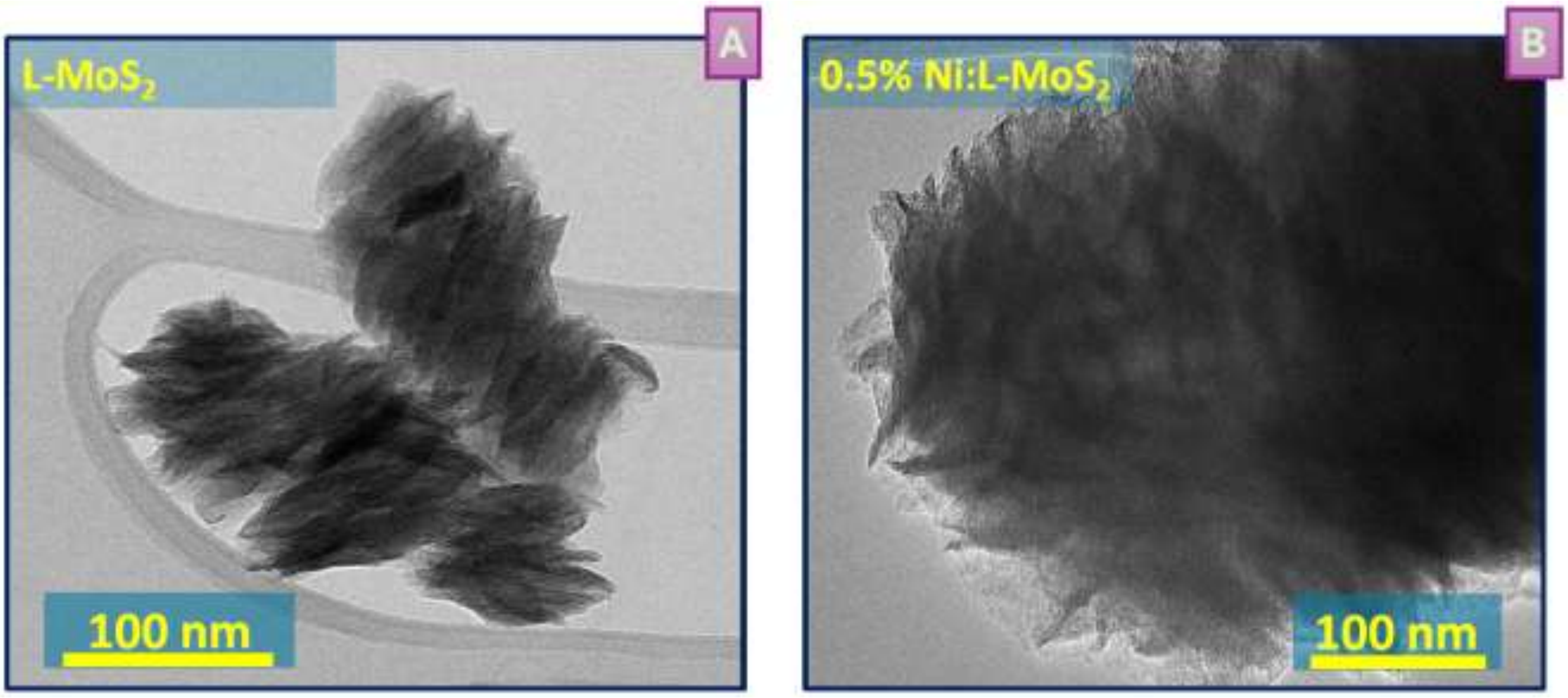

Figure S8. TEM images of chiral MoS_2 flakes with and without Ni doping (before delamination).

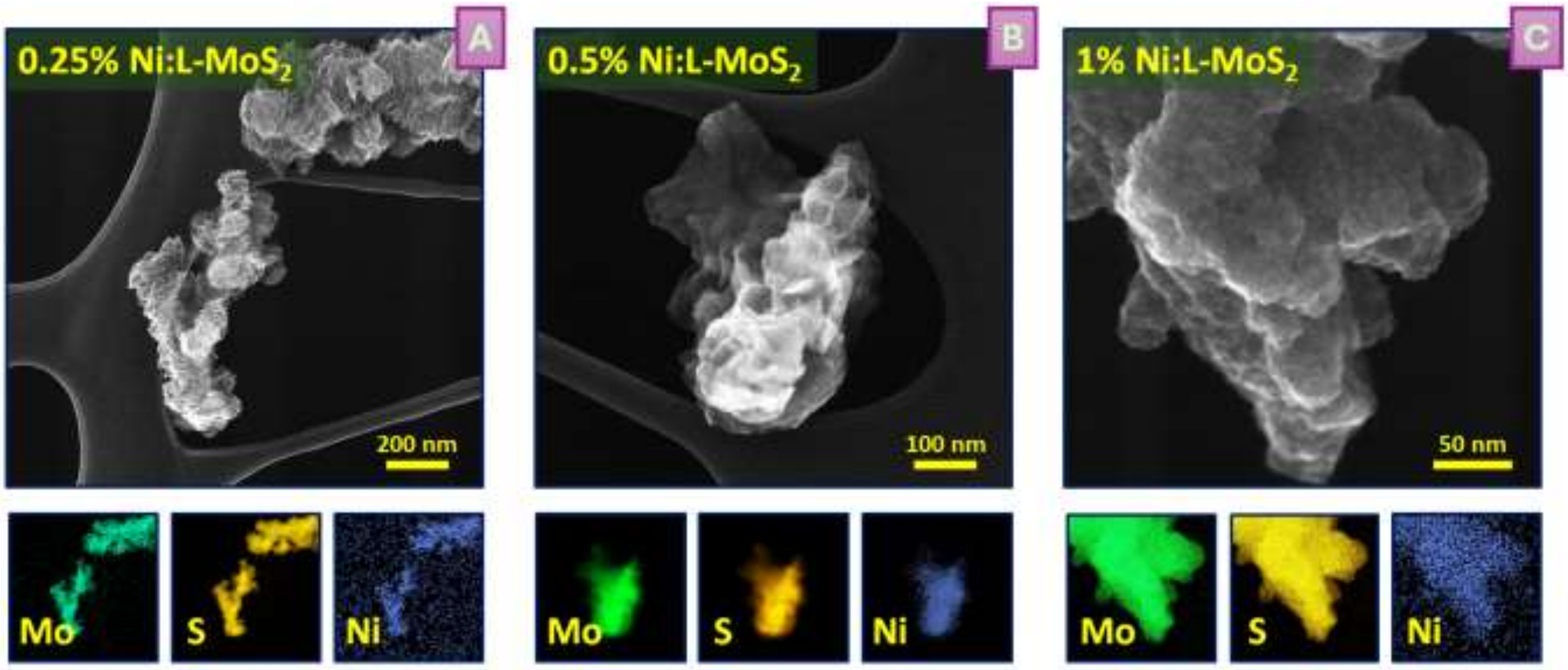

Figure S9. STEM SE images and EDX maps of as-prepared (non-delaminated) MoS_2 flakes with different Ni content.

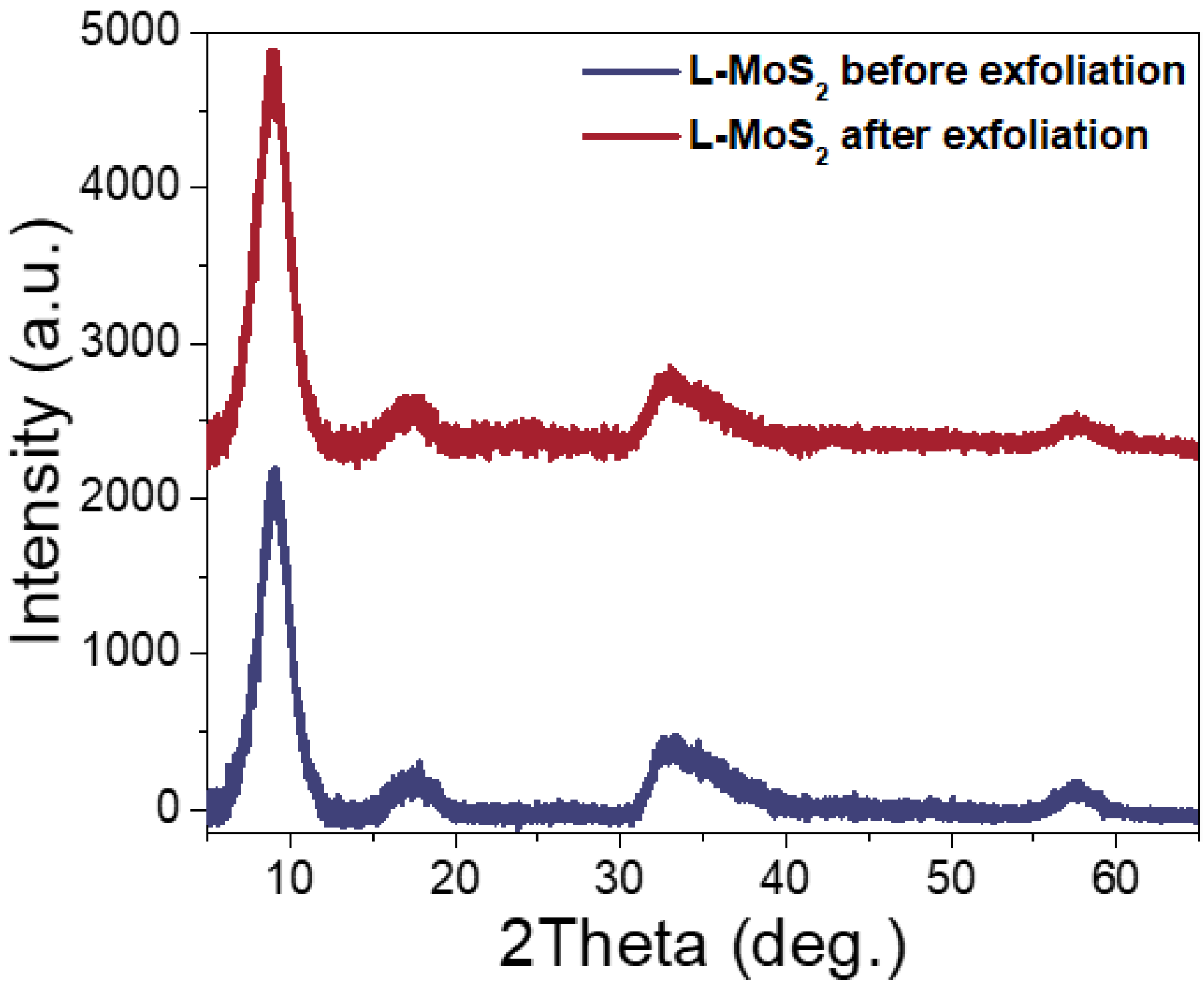

Figure S10. The XRD patterns of chiral MoS_2 without Ni addition, measured before and after exfoliation.

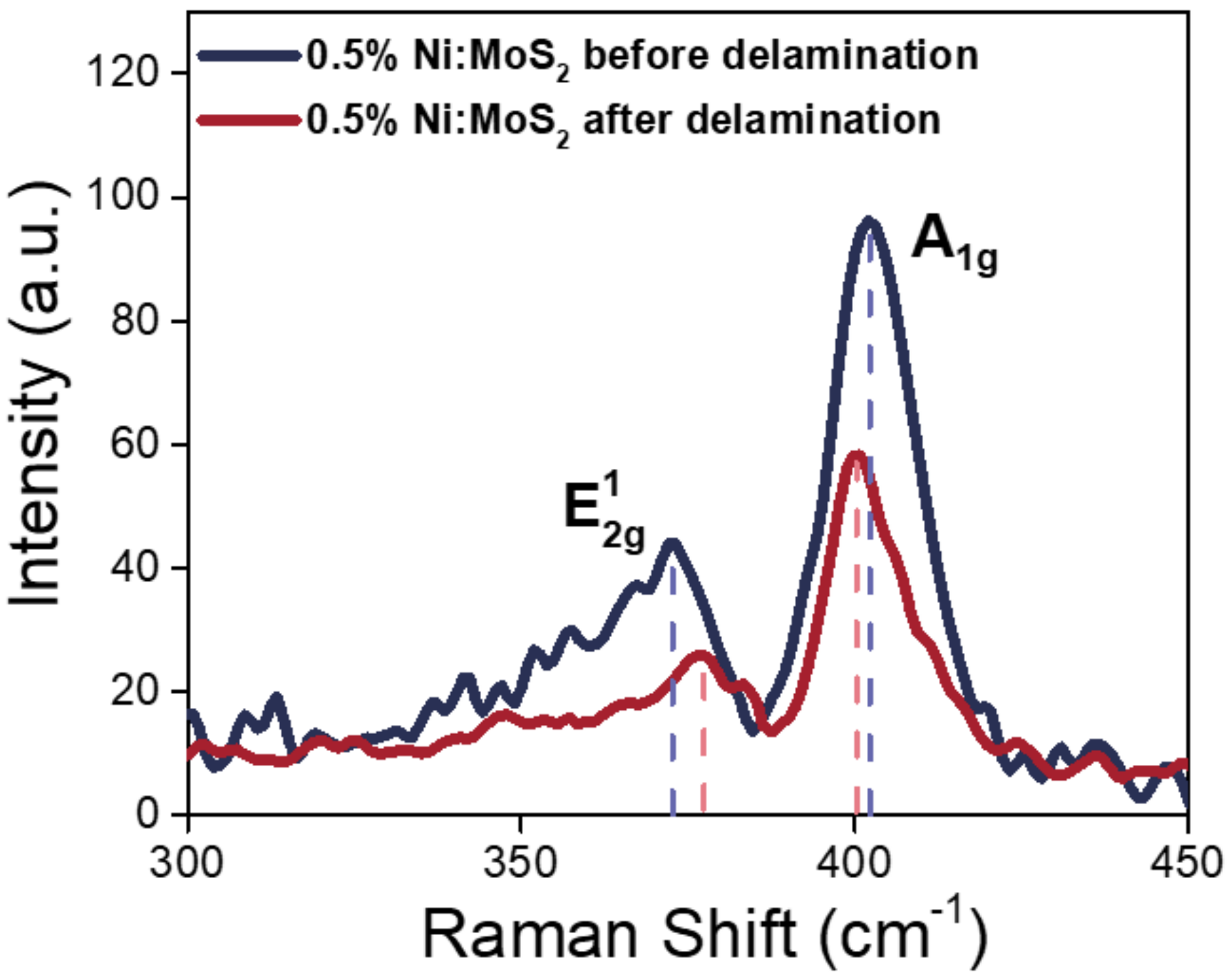

Figure S11. Raman spectra of achiral MoS_2 measured before and after delamination.

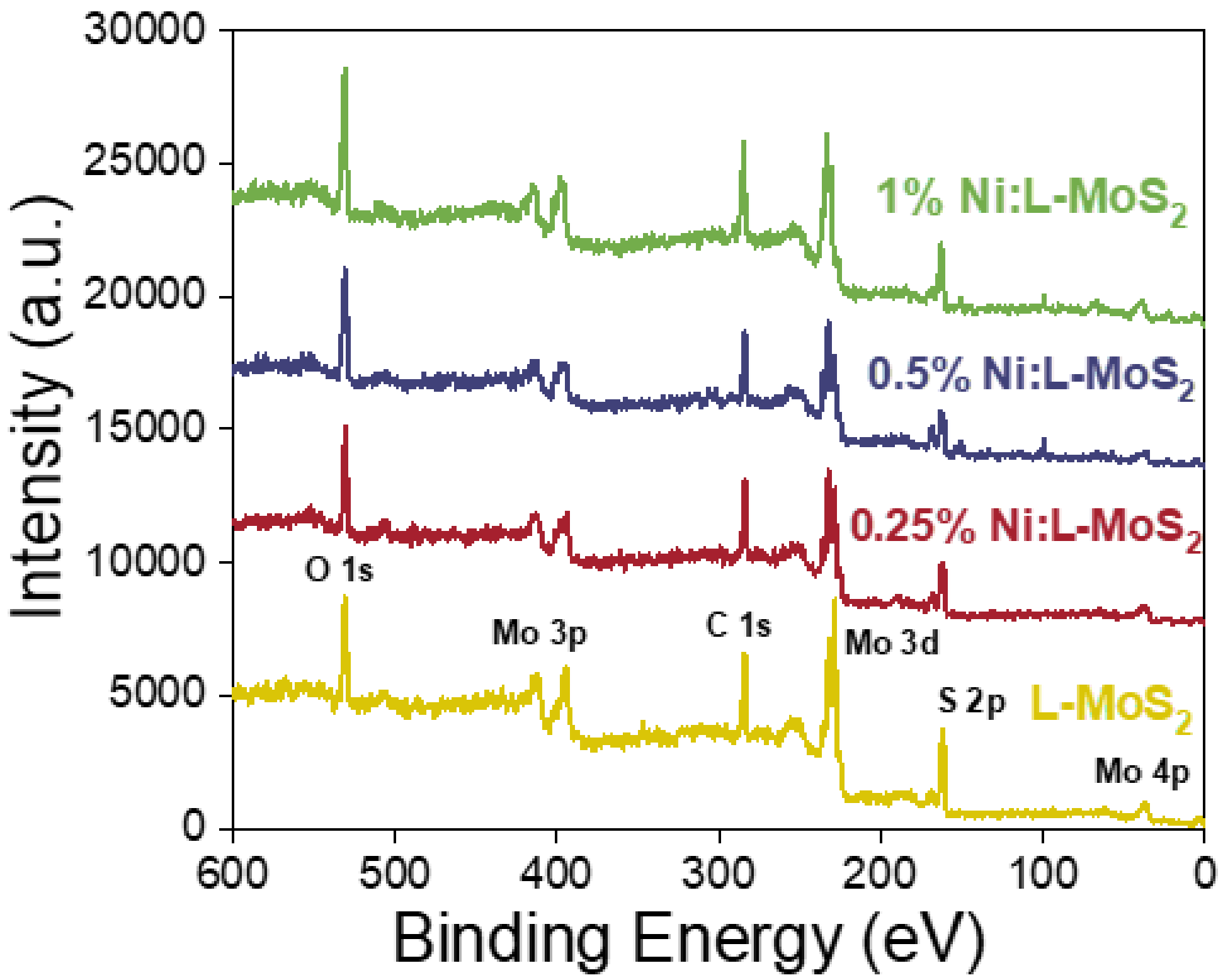

Figure S12. Survey spectra of chiral MoS_2 doped with different Ni amounts.

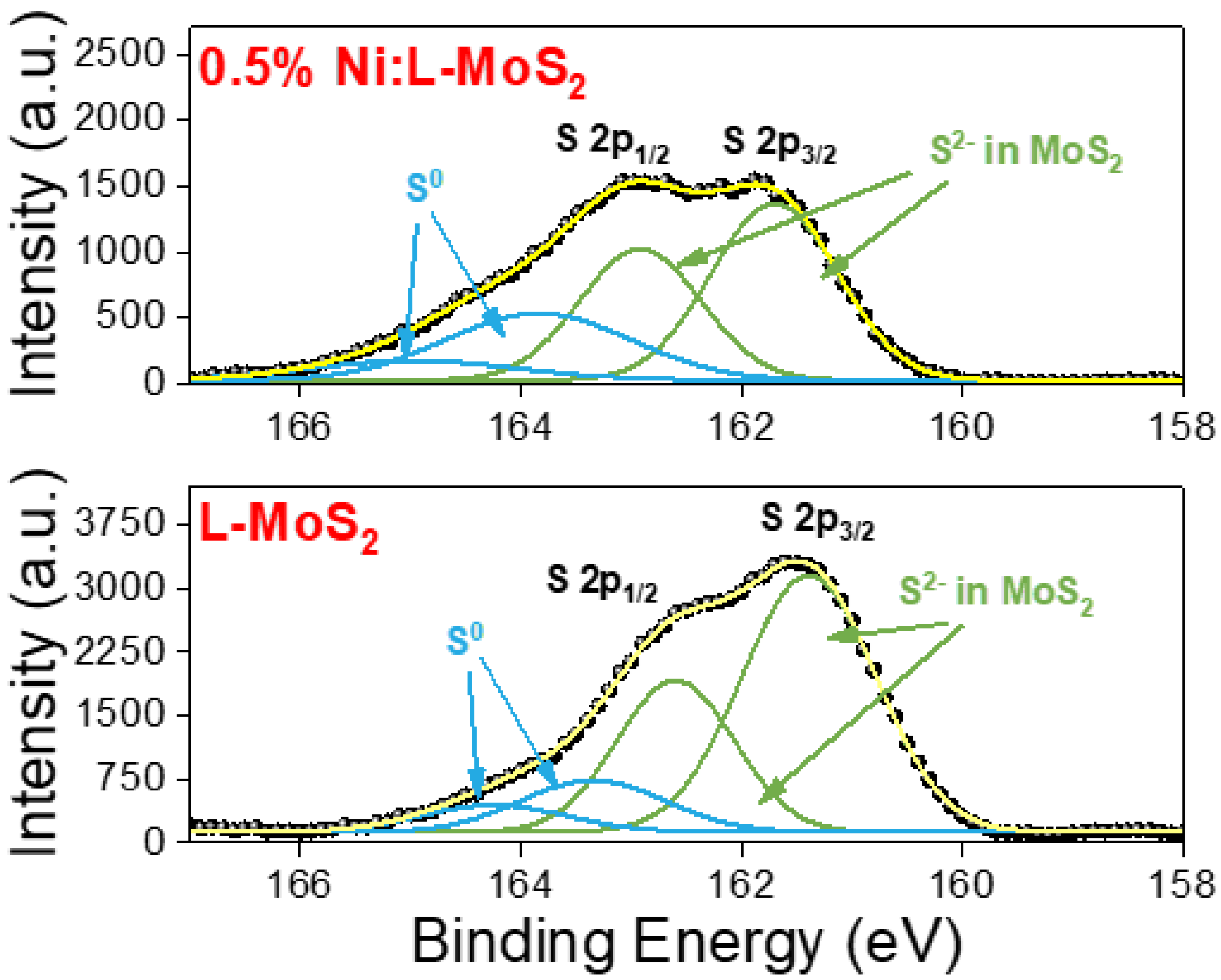

Figure S13. High resolution sulfur XPS spectrum, measured on the chiral flakes with and without Ni doping (0.5 %).

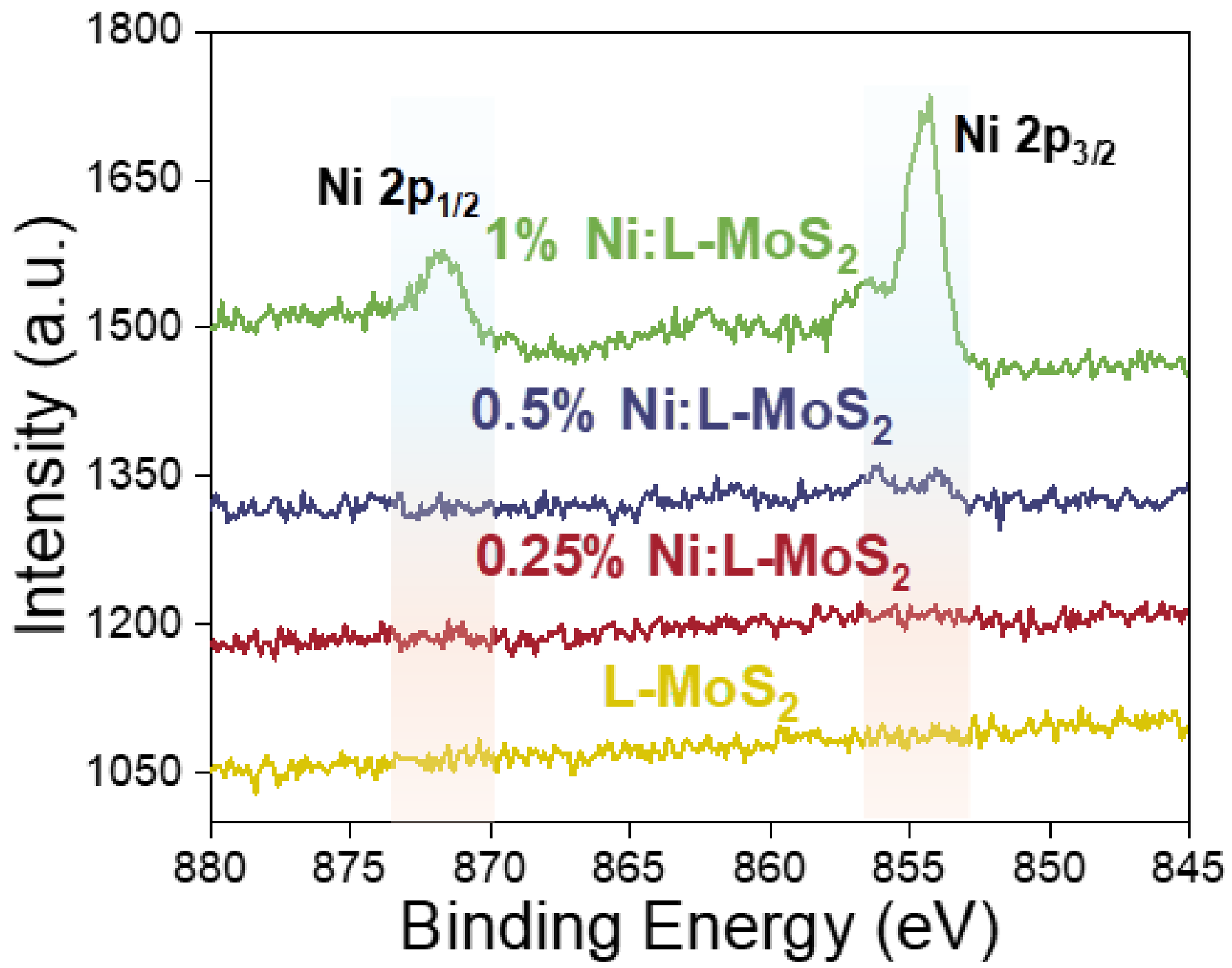

Figure S14. High resolution nickel XPS spectrum, measured on the chiral flakes as a function of Ni content.

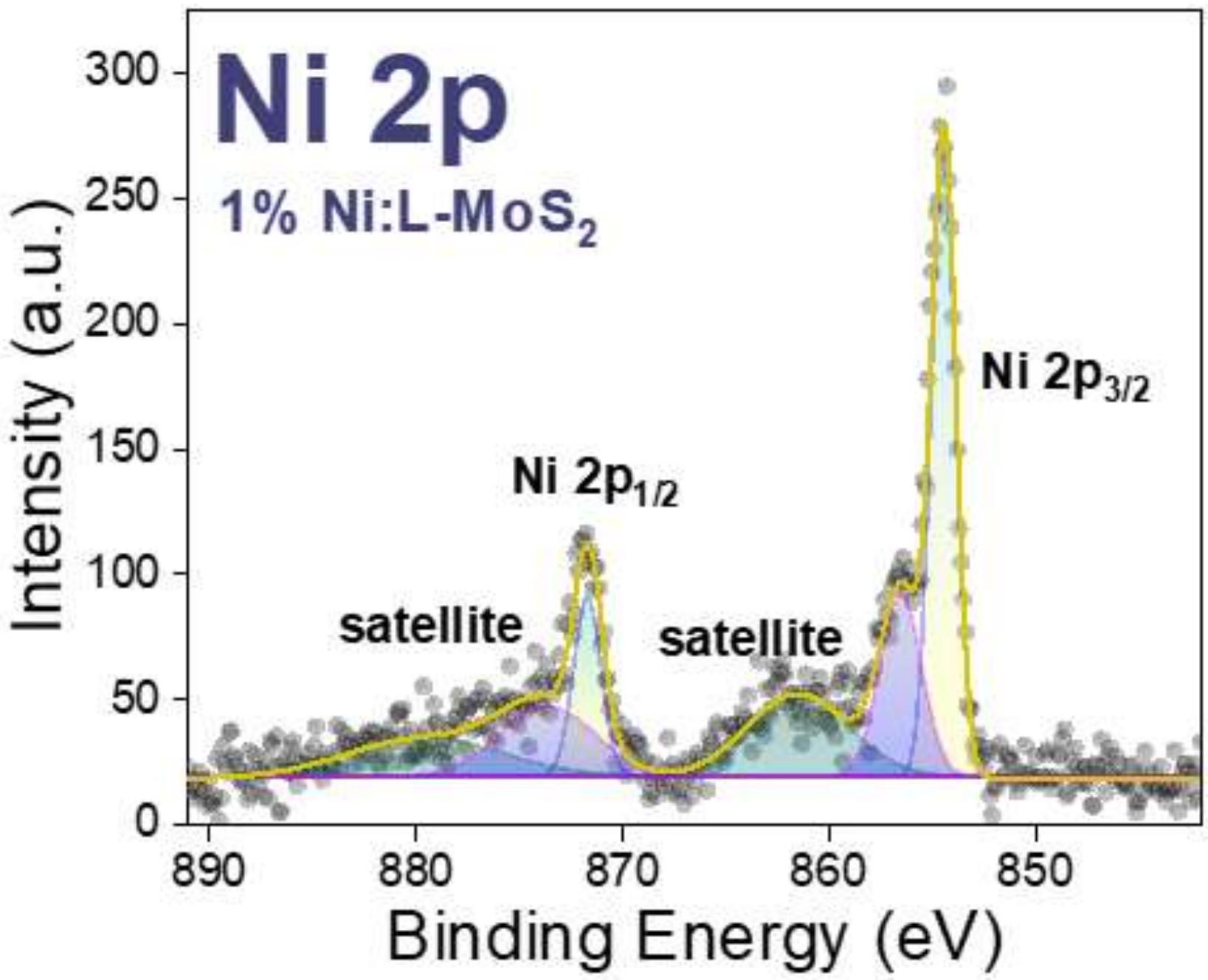

Figure S15. Deconvolution of high resolution XPR peak of Ni (chiral MoS_2 doped with 1.0 % of nickel).

Figure S15 – description note: To further understand type of Ni presented in MoS_2 structure, the deconvolution of the Ni 2p peak area for 1.0 % Ni:L-MoS_2 is shown in Fig. S14. Here, significantly split spin-orbit components (Δ = 17.0 eV) are clearly visible. The multiplicity of peaks can be explained by the presence of the oxide form of Ni (II).

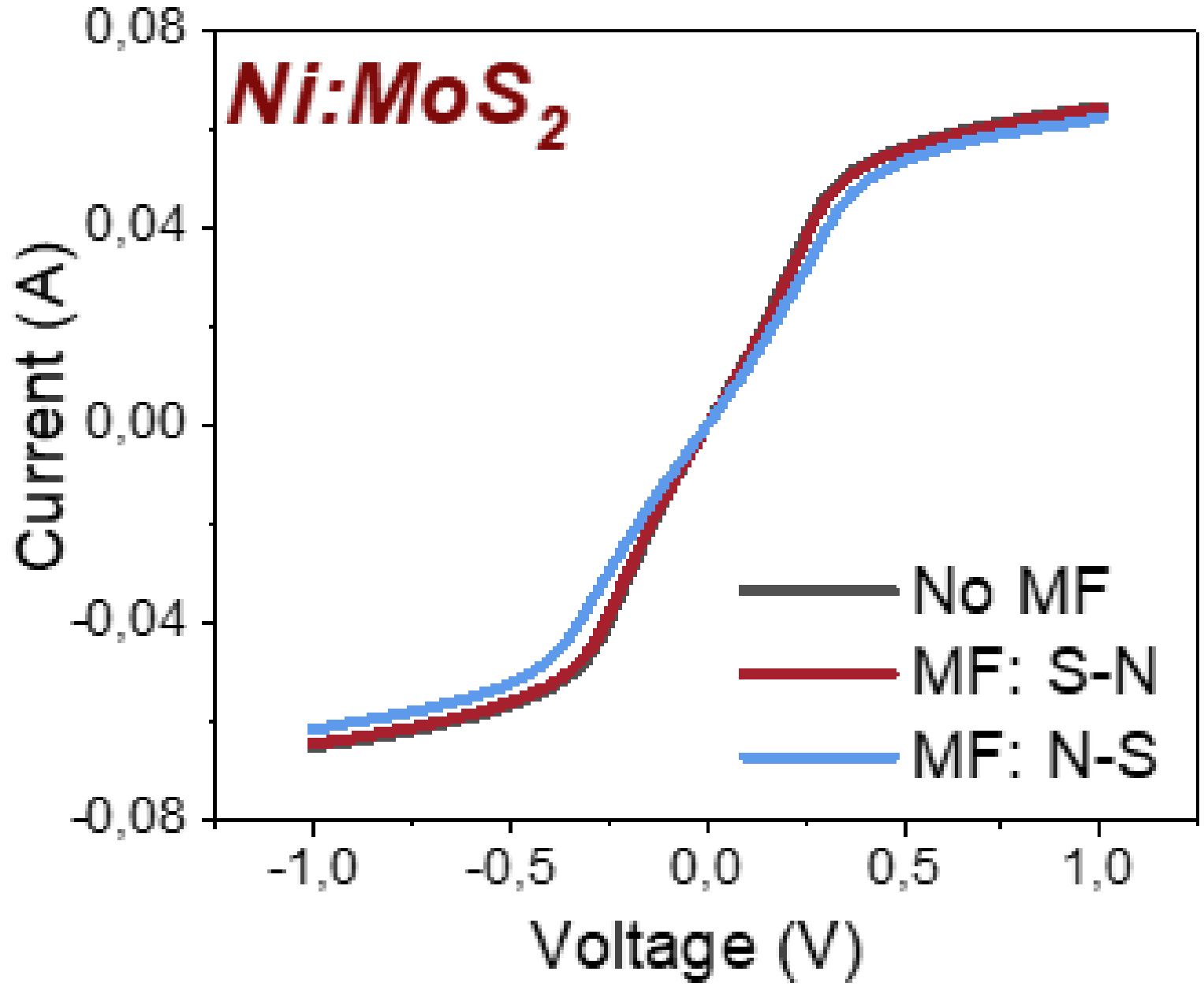

Figure S16. Mott polarimetry performed using the measurements of I-V curves across the achiral Ni-doped (prepared without TA addition) MoS_2//Ni//Au structure.

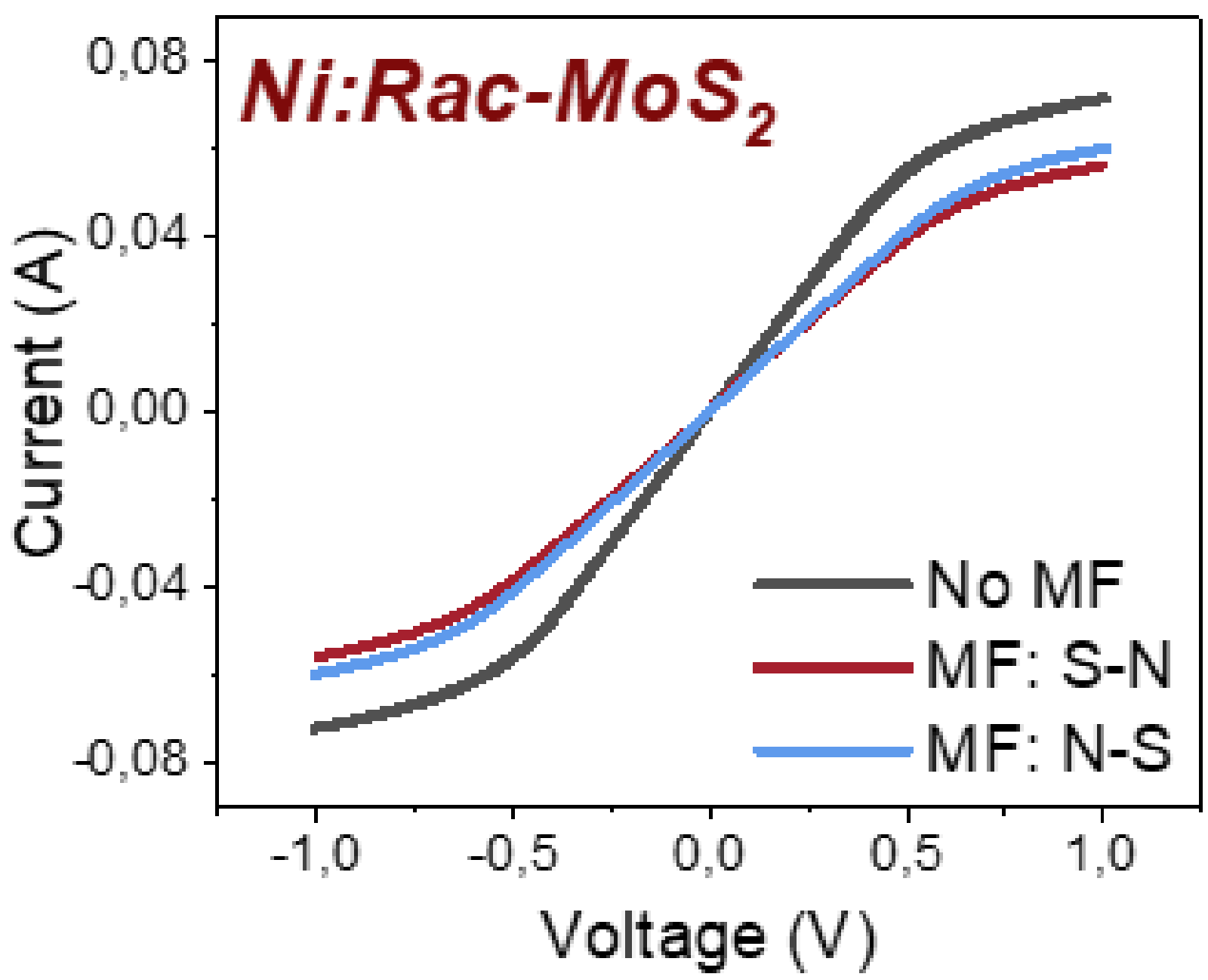

Figure S17. Mott polarimetry performed using the measurements of I-V curves across the achiral Ni-doped (prepared with addition of TA racemate) MoS_2//Ni//Au structure.

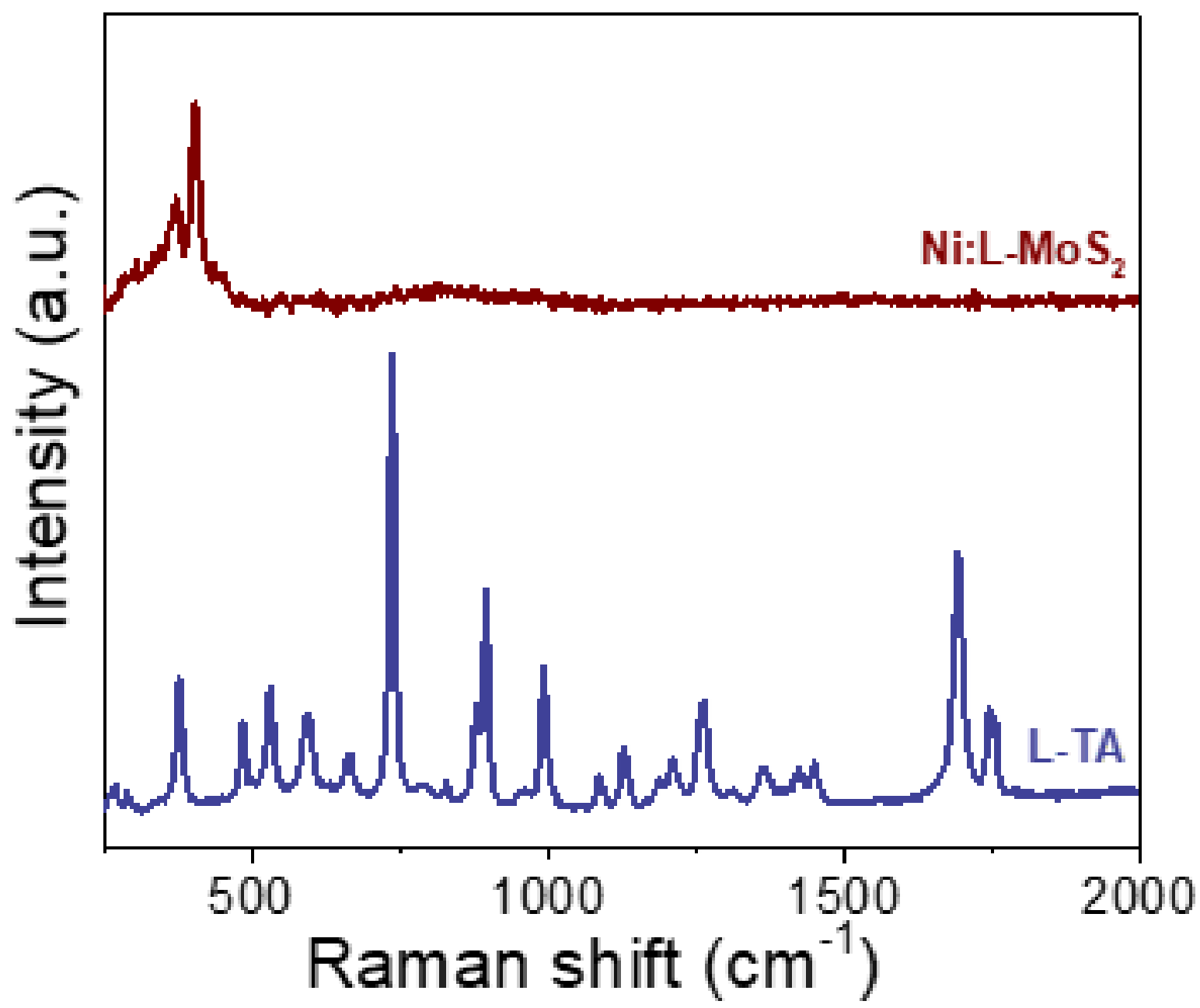

Figure S18. Raman spectra of TA acid powder and chiral, Ni-doped MoS_2 flakes. The absence of characteristic TA band is evident and indicates the fact, that the chirality origin cannot be attributed to organic molecules entrapment.

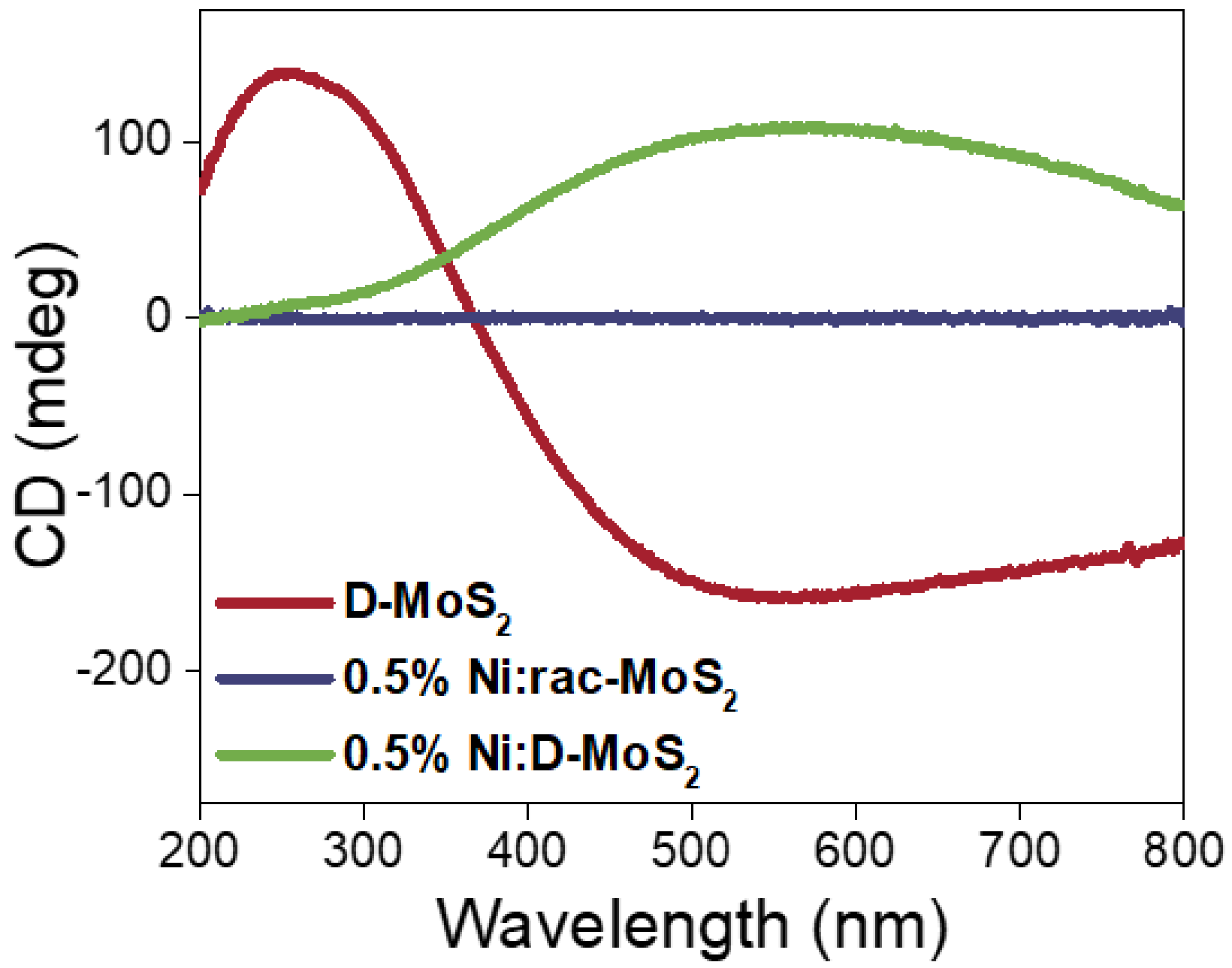

Figure S19. Solid state CD measurements of D-MoS_2 flakes with and without Ni doping (opposite chirality compared to Fig. 3).

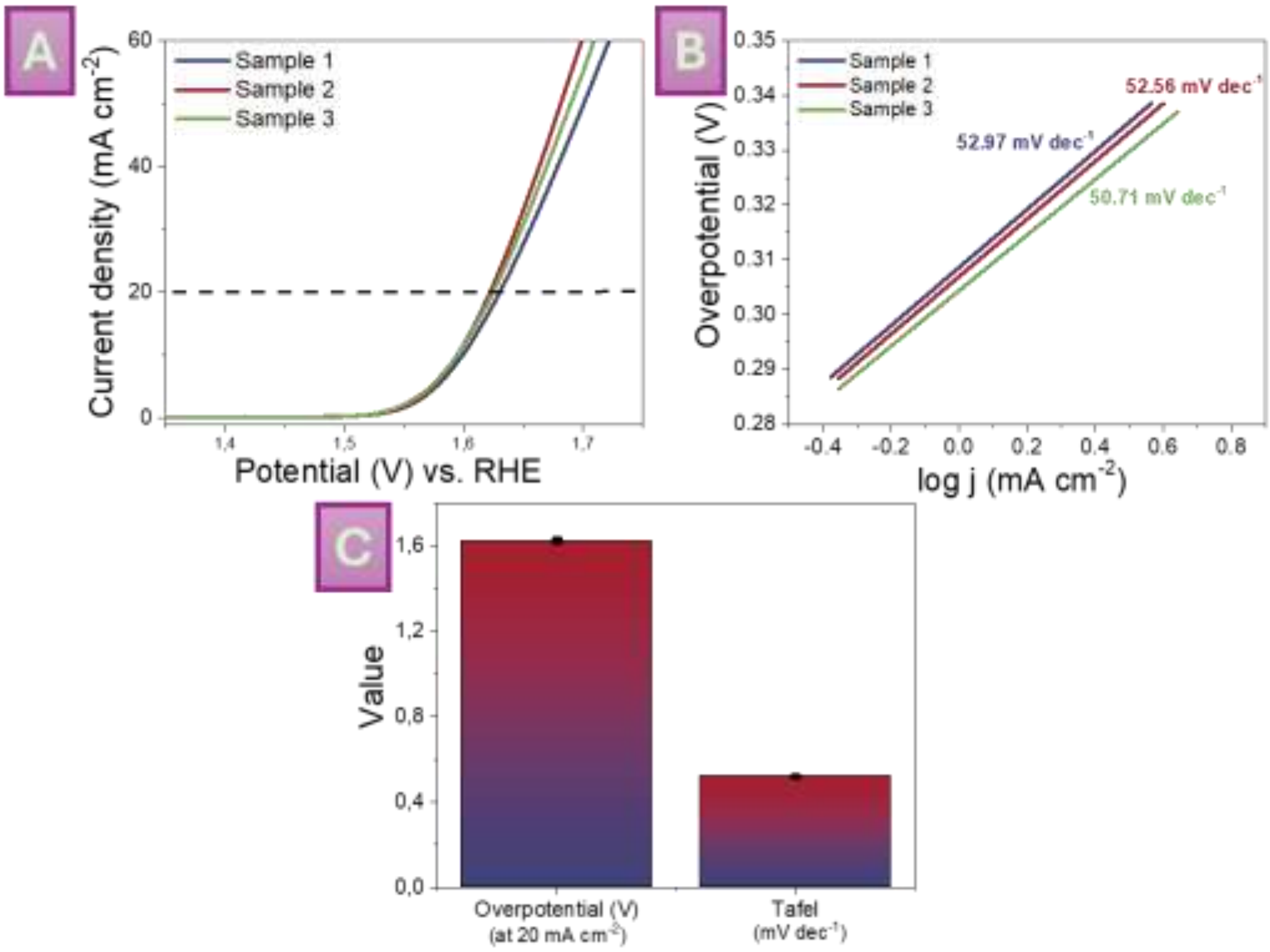

Figure S20. Statistical conformation of the reproducibility of electrochemical measurements: LSV curve and calculated values of Tafel slope. In Figures C, the error bars represent the mean ± SD (n=3).

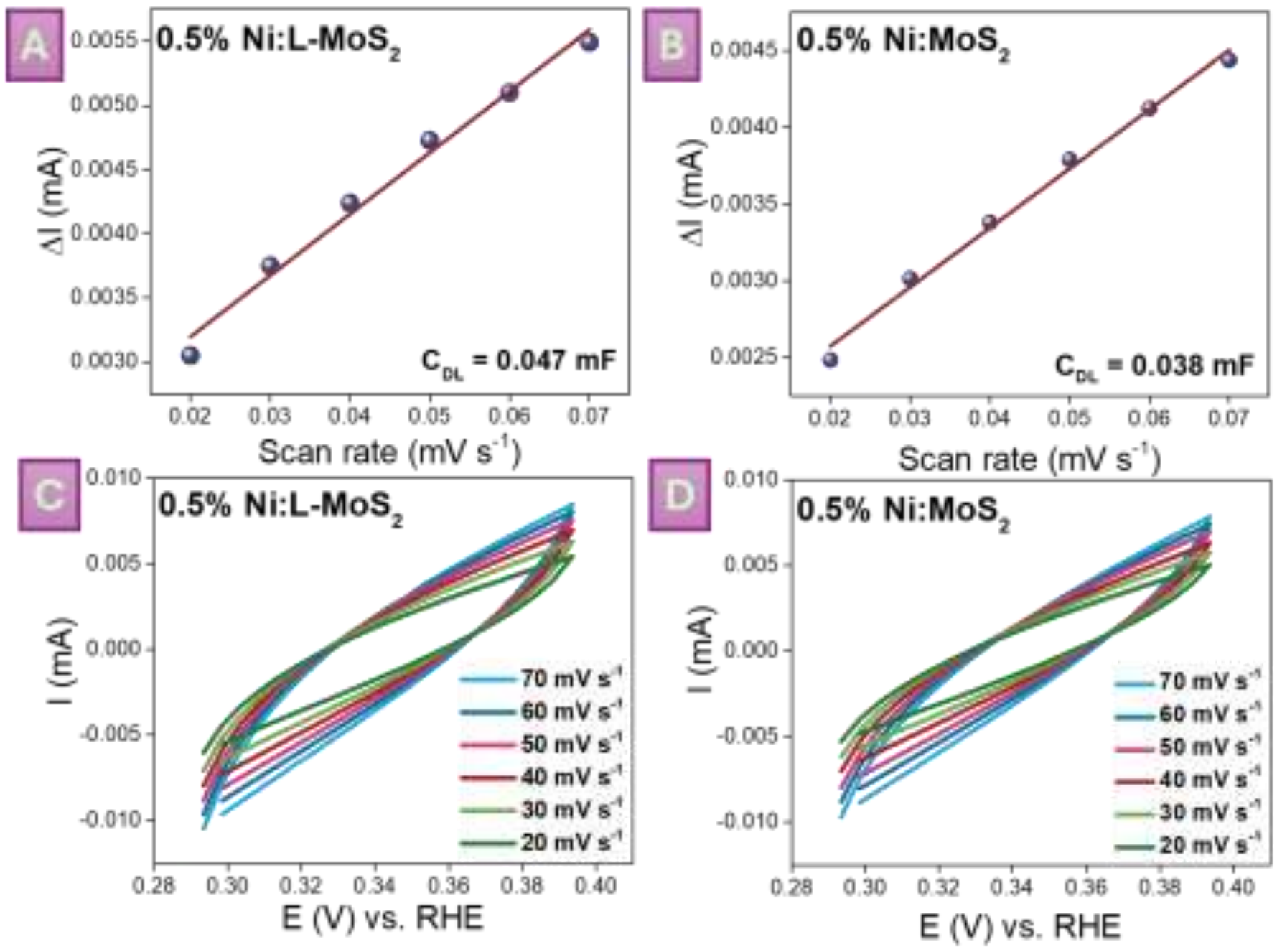

Figure S21. LSV curves measured at different scan rates and dependencies of Δ*I* on scan rate (subsequently used of ECSA calculation) for non-chiral (A, B) and chiral (C, D) doped flakes.

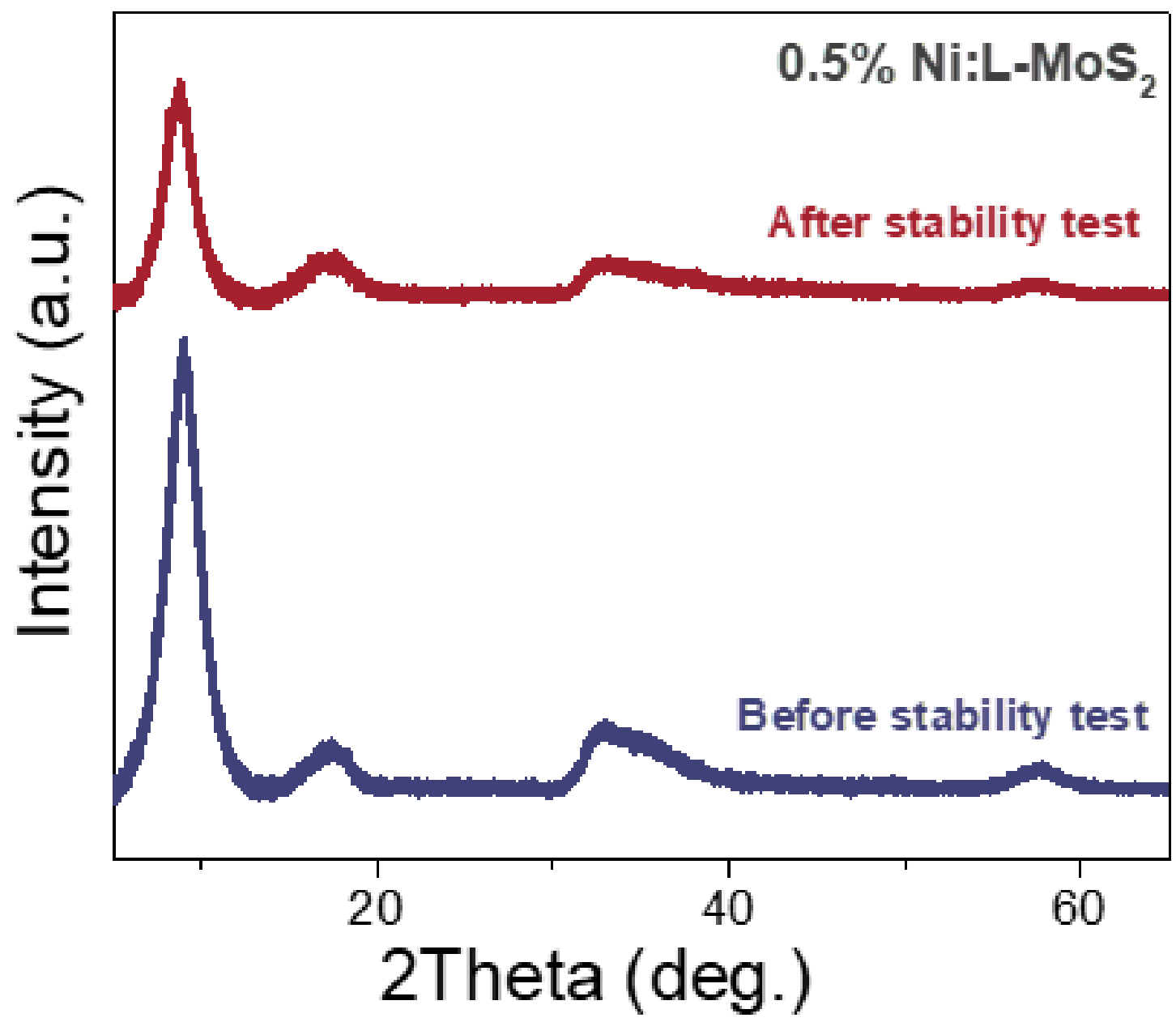

Figure S22. XRD spectra of chiral Ni:L-MoS_2, measured before and after stability tests.

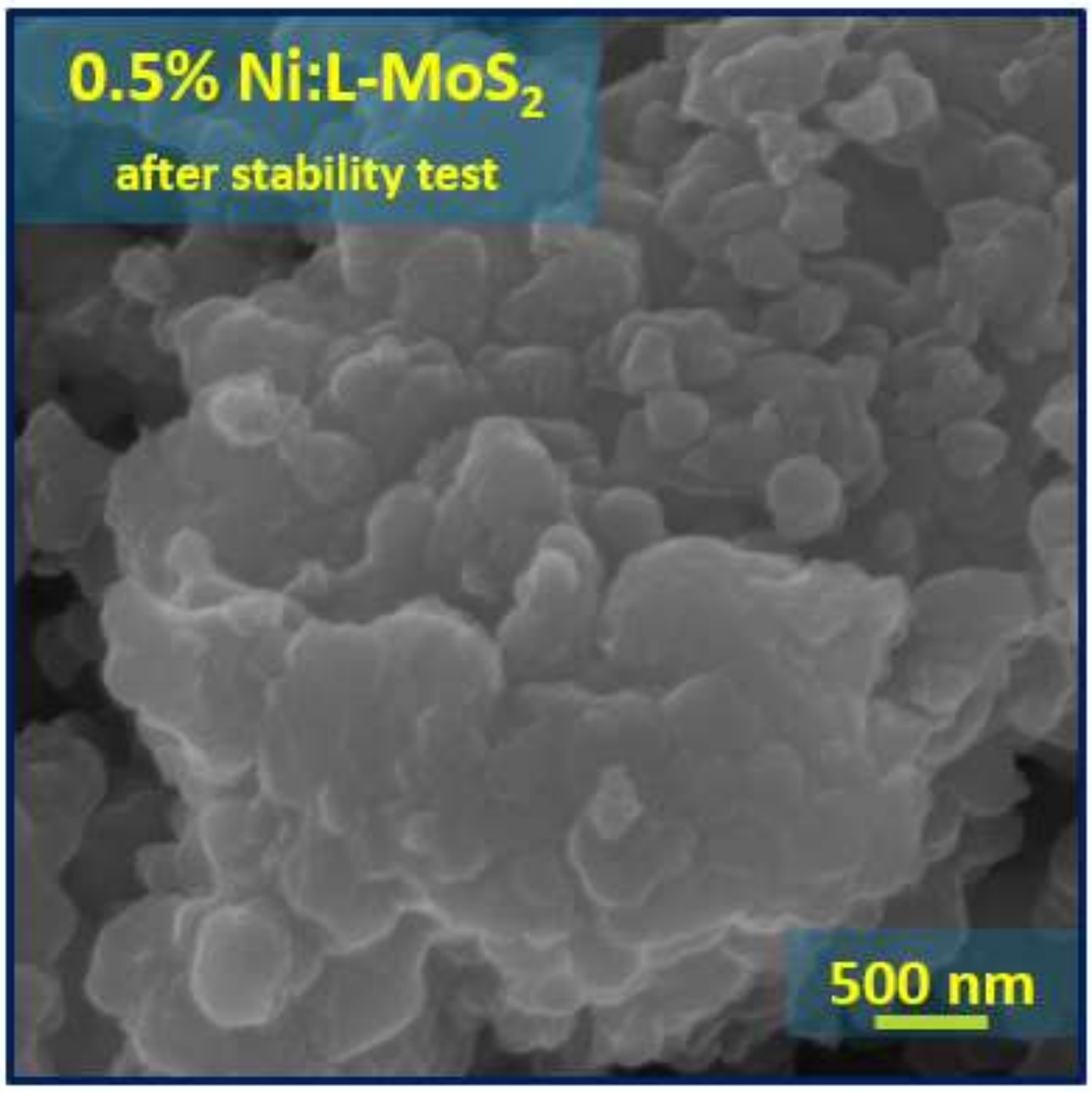

Figure S23. SEM measured morphology of chiral Ni:L-MoS_2 flakes after their utilisation in OER (after stability tests).

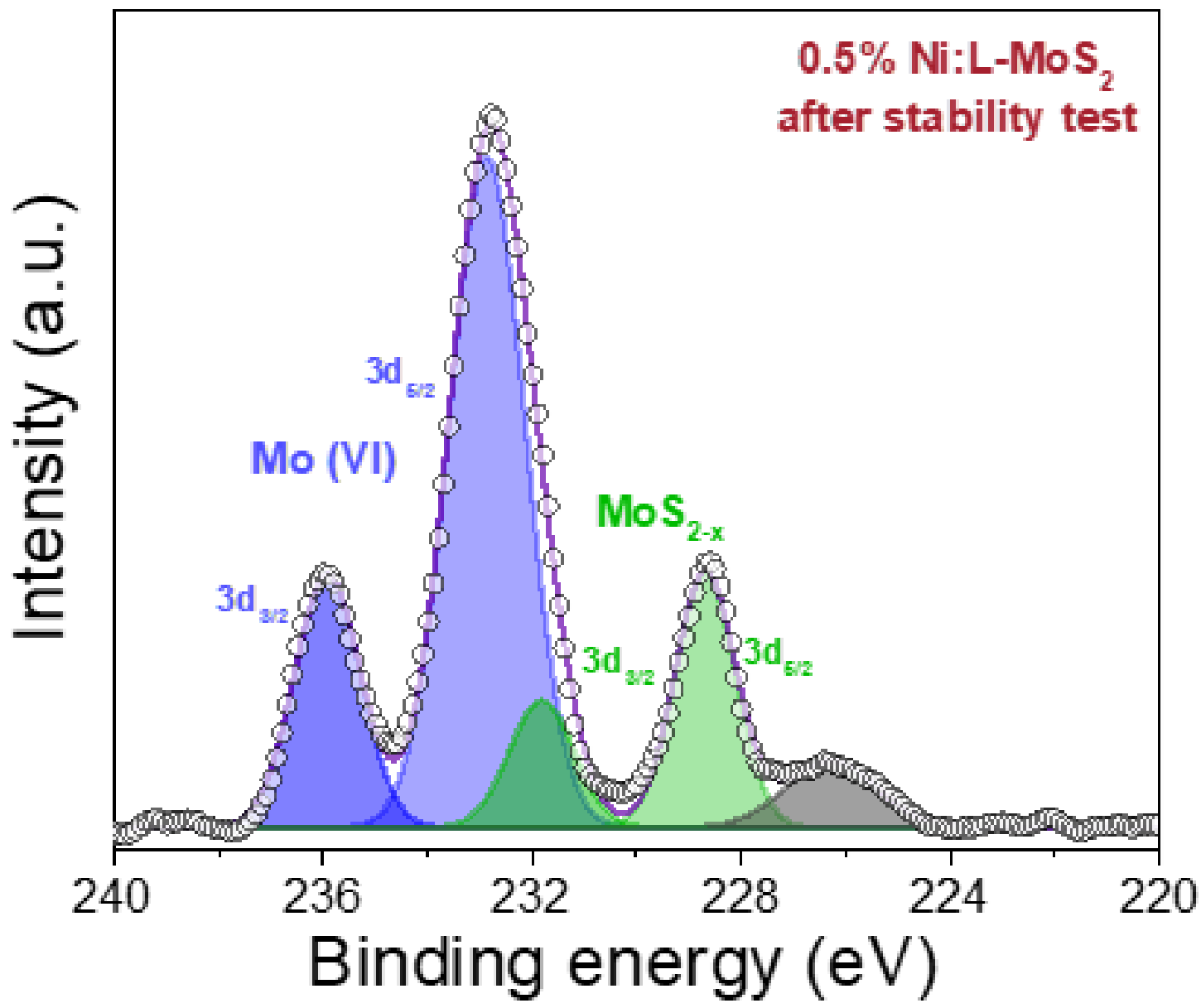

Figure S24. High resolution XPS scan of Mo-related spectral region.

Figure S24 – description note.

Following the reaction, an increase in oxide content on the surface was observed, indicating the formation of metal oxide species during the OER. Typically, the OER mechanism involves the formation of metal-oxygen (Me-O) bonds at the catalyst surface. Therefore, the presence of Mo (VI) species, as detected by XPS after the reaction, can be attributed to the formation of Mo-O bonds resulting from surface oxidation processes occurring under OER conditions.

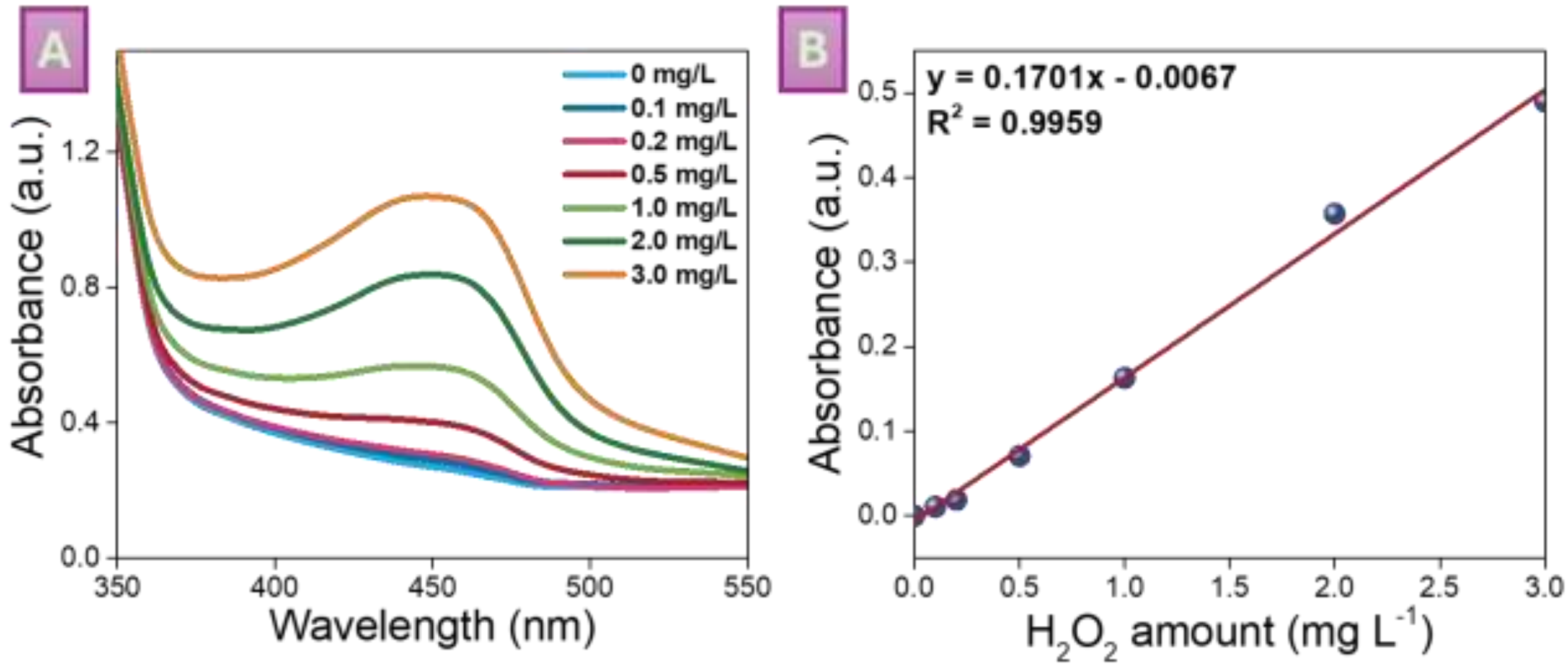

Figure S25. UV-vis absorption spectra (A) and calibration curve used for H_2O_2 detection (B).

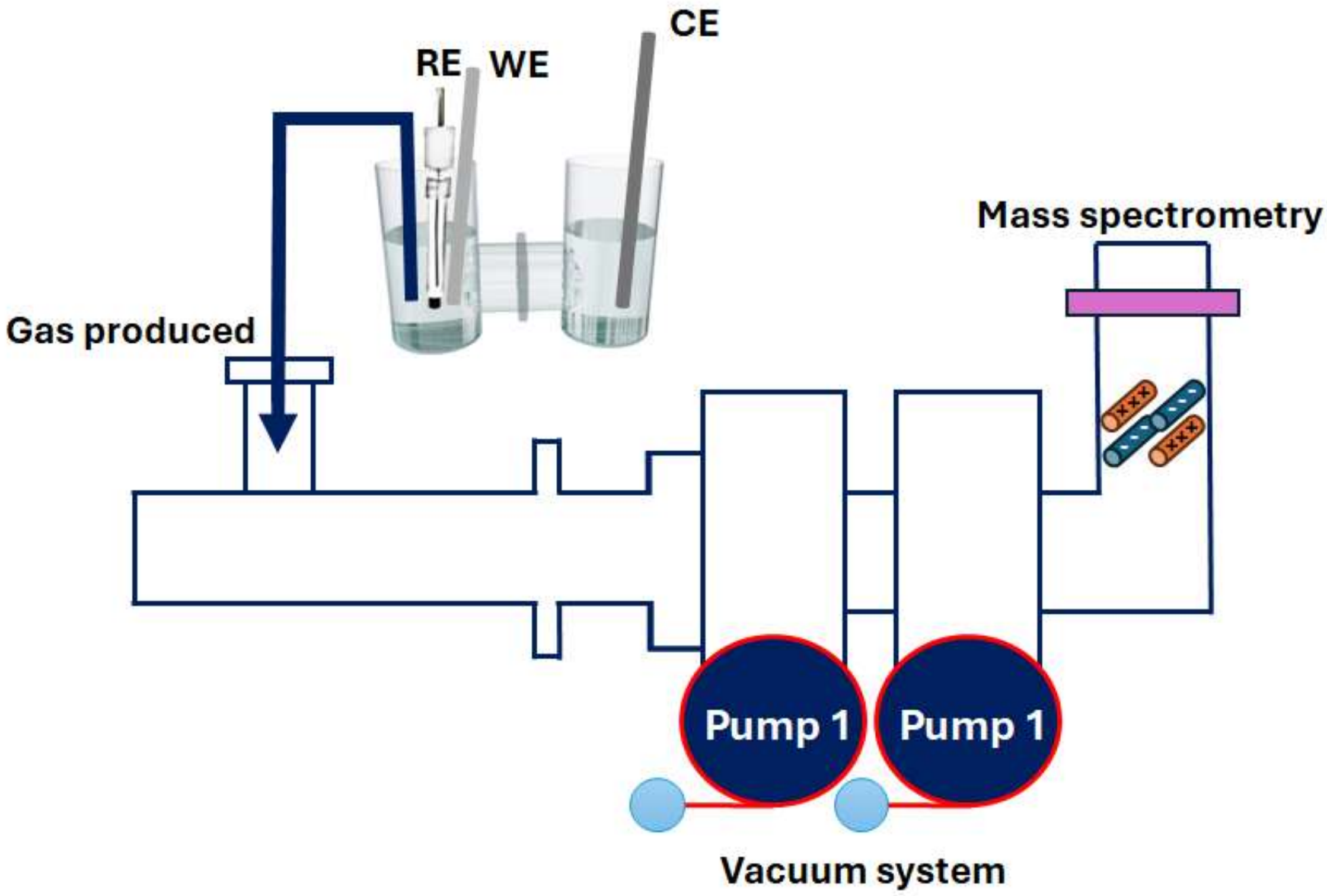

Figure S26. Schematic representation of DEMS experiments: electrochemical water splitting and reaction product analysis using in-operando sampling and measurements.

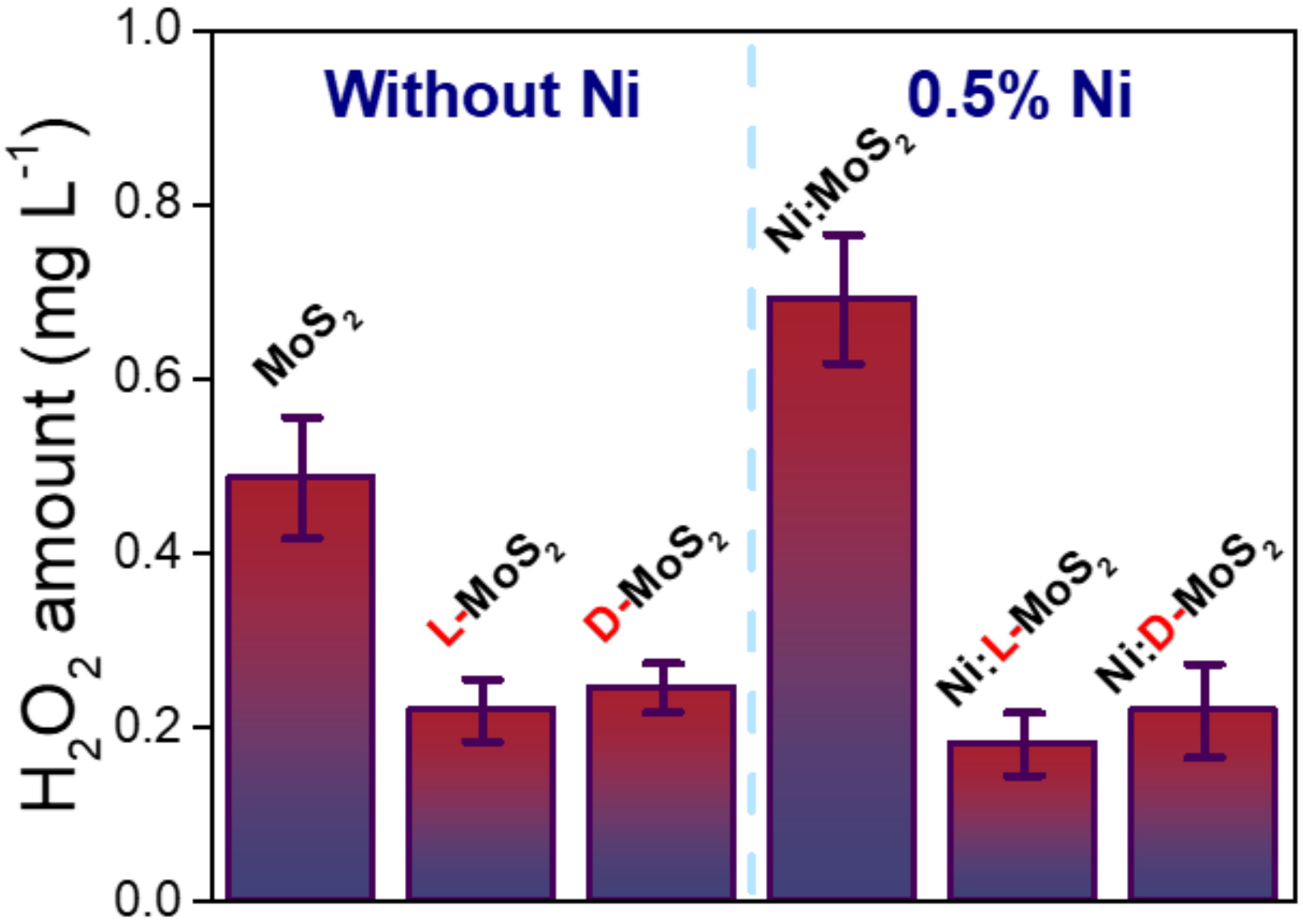

Figure S27. Non-normed production of H_2O_2 obtained with then use of chiral or achiral Ni doped or non-doped MoS_2 flakes (without considering the total amount of charge that has passed through the system). The error bars represent the mean ± SD (n=3)

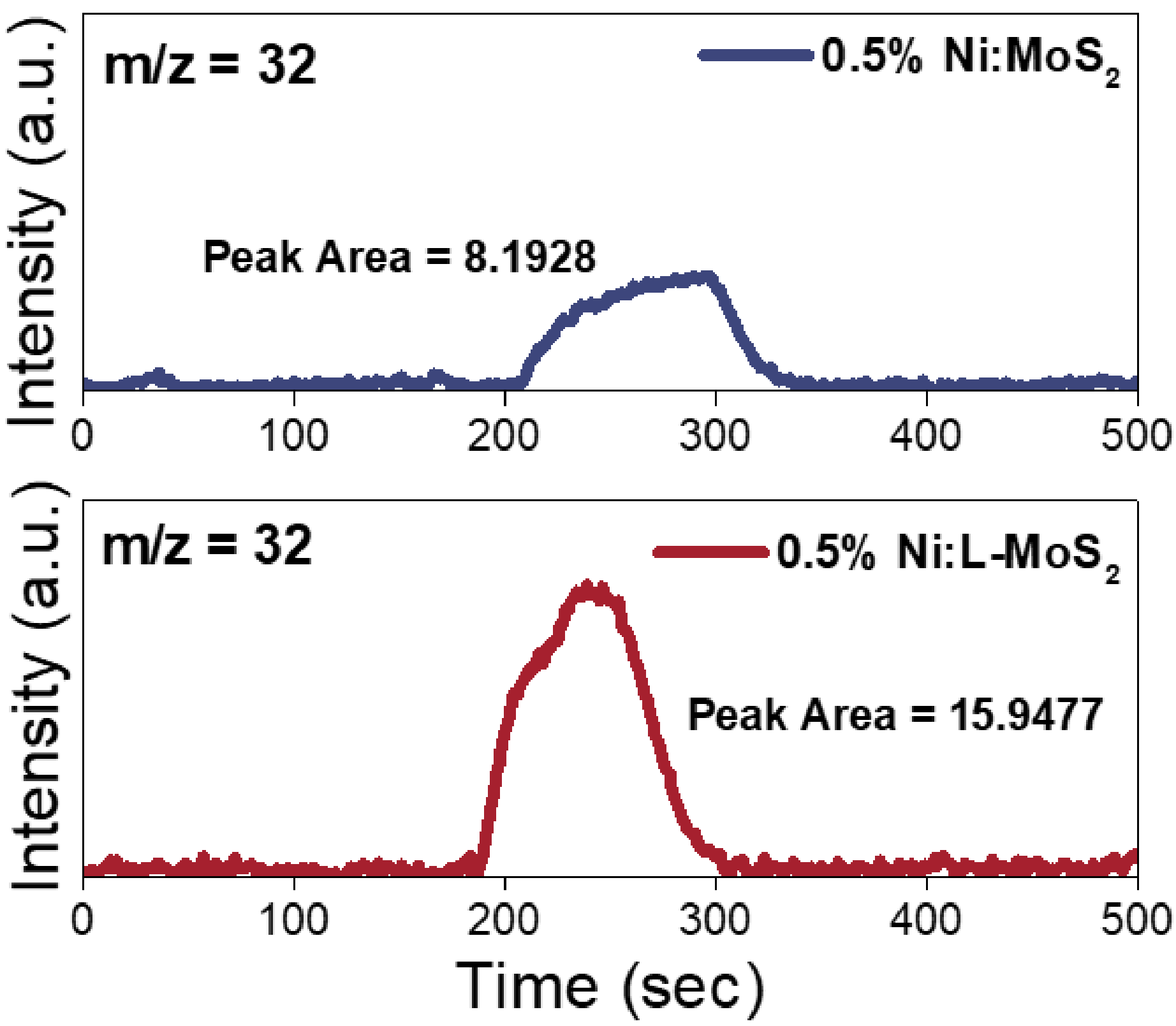

Figure S28. Characteristic oxygen related DEMS peak, measured after external bias switching.

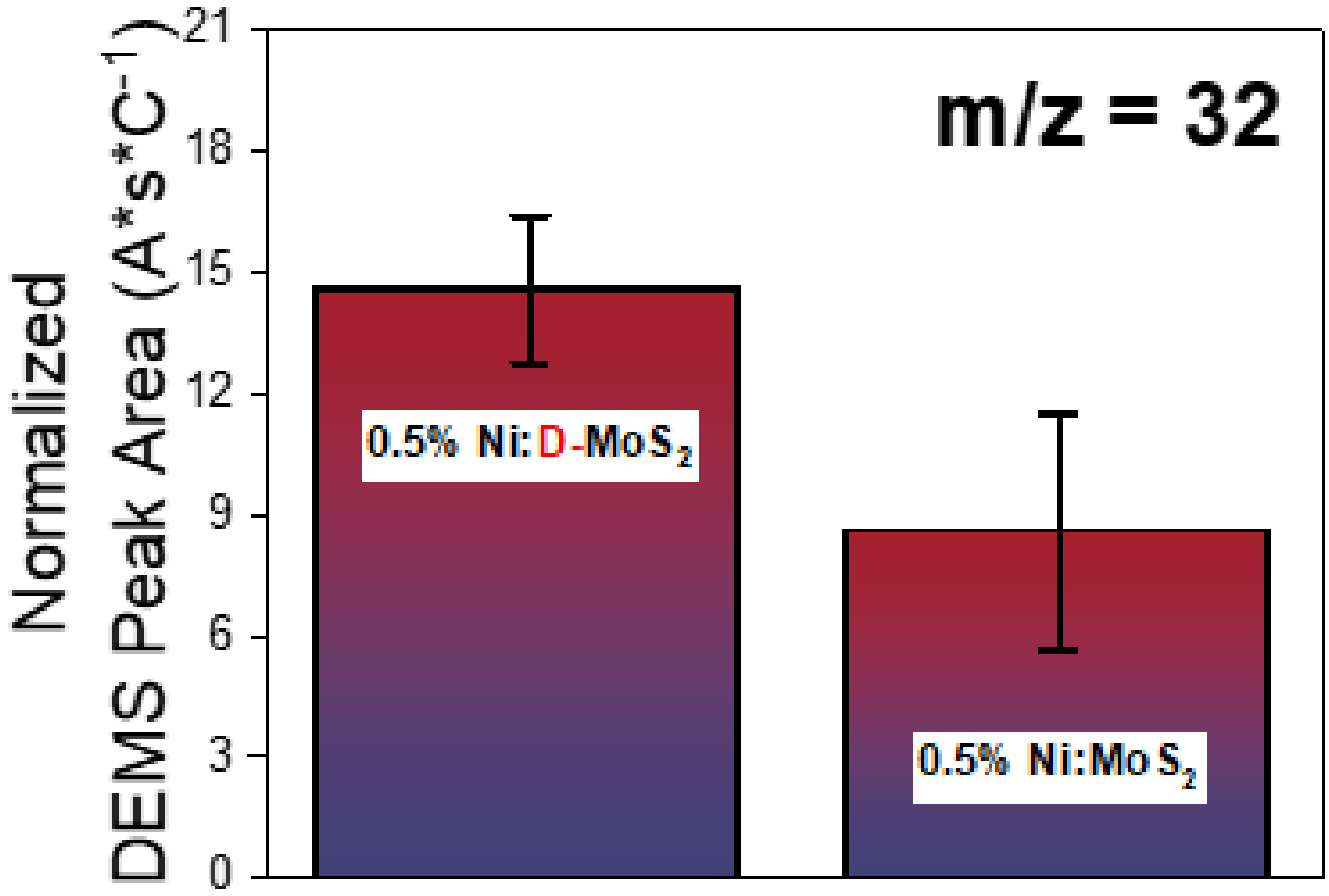

Figure S29. DEMS measured oxygen evolution on chiral and achiral flakes (normalized on the total charge consumed in the electrochemical cell during OER). The error bars represent the mean ± SD (n=3).

The DFT calculations were carried out with the CP2K package [S3]. The DFT calculations used the combination of the Gaussian and plane-wave (GPW) [S4] scheme for electronic density representation, double-ζ valence plus polarization (DZVP) basis sets of the MOLOPT [S5] type to describe the valence electrons and norm-conserving Goedecker-Teter-Hutter (GTH) [S6] pseudopotentials to approximate the core electrons. The generalized gradient approximation (GGA) of the Perdew−Burke−Ernzerhof (PBE) [S7] functional was used to describe the exchange-correlation interactions. DFT-D3 method [S8] was adopted to consider the van der Waals (vdW) interaction. The plane-wave basis set was truncated at the energy cutoff of 600 Ry. For wave function optimization, we used the orbital transformation (OT) method with the FULL_ALL preconditioner, a small energy gap value (0.005 a.u.), and the DIIS minimizer. The structures were optimized using a self-consistent field convergence threshold of 1E-7. For the MoS_2 flake (Fig. S30) description, we used bulk structure placed in 35×35×15 Å3 size box with periodic boundary conditions in the XY plane. We considered the OER in the following $2e^-$ and $4e^-$ reduction elementary steps (* denotes the active sites on the catalyst surface):

A) $2e^-$ reduction:

$$OH + * \rightarrow *OH + e^- \quad (1)$$

$$OH + *OH \rightarrow *OH\text{-}OH + e^- \quad (2)$$

B) 4e⁻ reduction:

OH + * → *OH + e^- (1)

OH+ *OH → *O + e^- (2)

OH+ *O → *OOH + e^- (3)

OH+ *OOH → * + O + H_2O + e^- (4)

The free energy of three intermediate states, *OH, *O, and *OOH, are used to identify OER activity. To calculate the free energy profiles of the OER, we used the computational hydrogen electrode (CHE) model:

$$\Delta G = \Delta E + \Delta E(ZPE) - T\Delta S,$$

where ΔE is the absorption energy, $\Delta E(ZPE)$ is the zero-point energy difference, and $T\Delta S$ is the entropy difference between the hydrogenation steps. For adsorbates, ZPE and S are determined by vibrational frequencies calculations. For molecules, those are taken from the NIST database [S9].

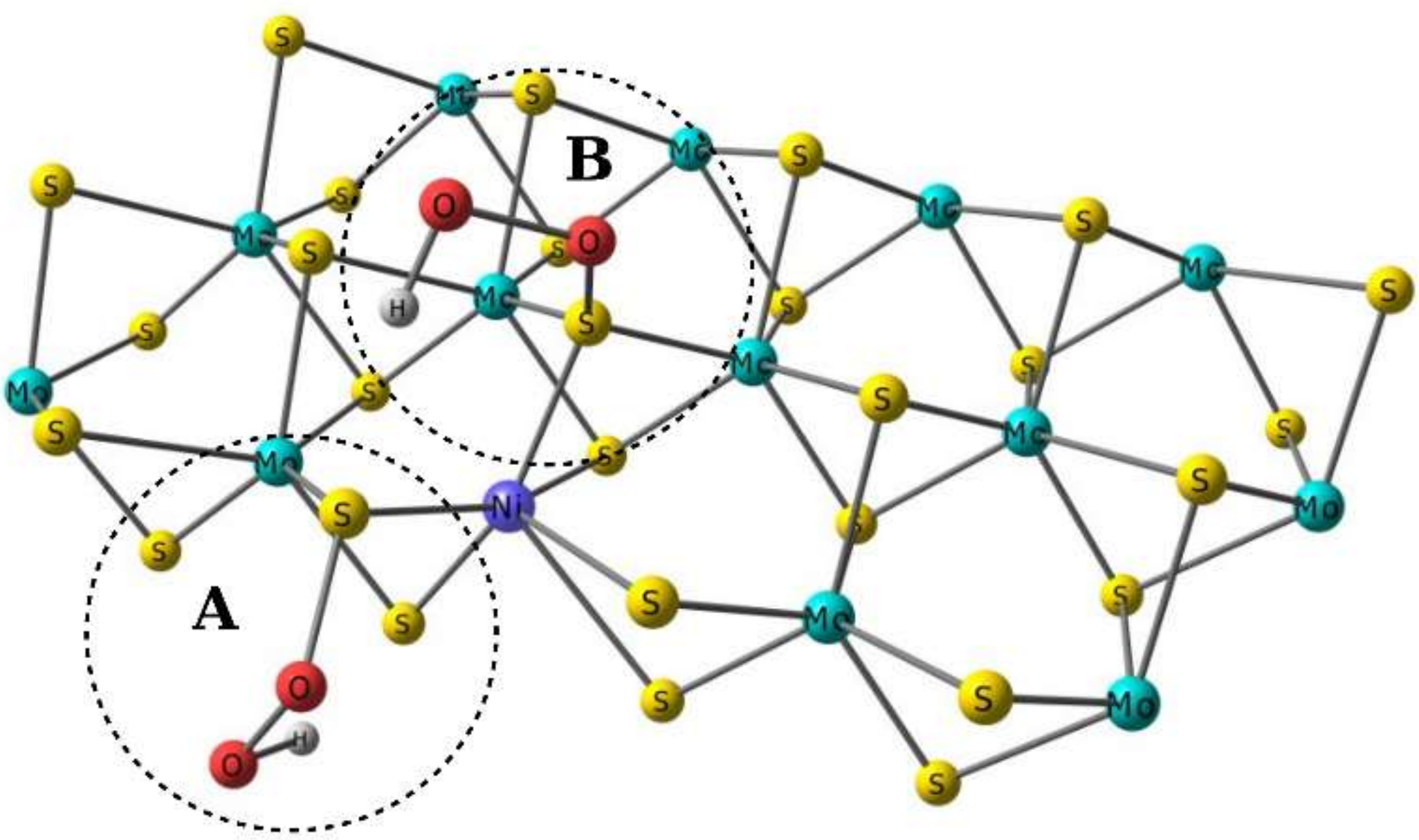

Figure S30. Illustration of the studied nickel-doped MoS_2 flake fragment with different active sites A and B.

References

[S1] L. Branzi, L. Fitzsimmons, Y. K. Gun'ko, *Angew. Chem. Int. Ed.* **2024**, *63*, e202409313.

[S2] L. Branzi, L. Fitzsimmons, I. Chunin, I. Shvets, Y. K. Gun'ko, *Angew. Chem.* **2025**, *137*, e202420437.

[S3] J. Hutter, M. Iannuzzi, F. Schiffmann, J. VandeVondele, *WIREs Comput. Mol. Sci.* **2014**, *4*, 15.

[S4] B. G. Lippert, J. H. and M. Parrinello, *Mol. Phys.* **1997**, *92*, 477.

[S5] J. VandeVondele, J. Hutter, *J. Chem. Phys.* **2007**, *127*, 114105.

[S6] S. Goedecker, M. Teter, J. Hutter, *Phys. Rev. B* **1996**, *54*, 1703.

[S7] J. P. Perdew, K. Burke, M. Ernzerhof, *Phys. Rev. Lett.* **1996**, *77*, 3865.

[S8] E. Caldeweyher, C. Bannwarth, S. Grimme, *J. Chem. Phys.* **2017**, *147*, 034112.

[S9] Johnson, R. Computational Chemistry Comparison and Benchmark Database, NIST Standard Reference Database 101, 2002.
